# *Chloroflexus aurantiacus* acetyl-CoA carboxylase evolves fused biotin carboxylase and biotin carboxyl carrier protein to complete carboxylation activity

**DOI:** 10.1128/mbio.03414-23

**Published:** 2024-04-04

**Authors:** Jiejie Shen, Wenping Wu, Kangle Wang, Jingyi Wu, Bing Liu, Chunyang Li, Zijun Gong, Xin Hong, Han Fang, Xingwei Zhang, Xiaoling Xu

**Affiliations:** 1 Department of Biochemistry and Molecular Biology, School of Basic Medical Sciences, Hangzhou Normal University, Hangzhou, China; 2 Zhejiang Key Laboratory of Medical Epigenetics, Hangzhou Normal University, Hangzhou, China; 3 The Affiliated Hospital of Hangzhou Normal University, Hangzhou Normal University, Hangzhou, China; 4 Photosynthesis Research Center, College of Life and Environmental Sciences, Hangzhou Normal University, Hangzhou, China; University of Washington School of Medicine, Seattle, Washington, USA; Columbia University, New York, New York, USA

**Keywords:** acetyl-CoA carboxylase, biotin carboxylase, biotin carboxyl carrier protein, carboxyltransferase, 3-hydroxypropionate

## Abstract

Acetyl-CoA carboxylases (ACCs) convert acetyl-CoA to malonyl-CoA, a key step in fatty acid biosynthesis and autotrophic carbon fixation pathways. Three functionally distinct components, biotin carboxylase (BC), biotin carboxyl carrier protein (BCCP), and carboxyltransferase (CT), are either separated or partially fused in different combinations, forming heteromeric ACCs. However, an ACC with fused BC-BCCP and separate CT has not been identified, leaving its catalytic mechanism unclear. Here, we identify two BC isoforms (BC1 and BC2) from *Chloroflexus aurantiacus*, a filamentous anoxygenic phototroph that employs 3-hydroxypropionate (3-HP) bi-cycle rather than Calvin cycle for autotrophic carbon fixation. We reveal that BC1 possesses fused BC and BCCP domains, where BCCP could be biotinylated by *E. coli* or *C. aurantiacus* BirA on Lys553 residue. Crystal structures of BC1 and BC2 at 3.2 Å and 3.0 Å resolutions, respectively, further reveal a tetramer of two BC1-BC homodimers, and a BC2 homodimer, all exhibiting similar BC architectures. The two BC1-BC homodimers are connected by an eight-stranded β-barrel of the partially resolved BCCP domain. Disruption of β-barrel results in dissociation of the tetramer into dimers in solution and decreased biotin carboxylase activity. Biotinylation of the BCCP domain further promotes BC1 and CTβ-CTα interactions to form an enzymatically active ACC, which converts acetyl-CoA to malonyl-CoA *in vitro* and produces 3-HP via co-expression with a recombinant malonyl-CoA reductase in *E. coli* cells. This study revealed a heteromeric ACC that evolves fused BC-BCCP but separate CTα and CTβ to complete ACC activity.

**IMPORTANCE:**

Acetyl-CoA carboxylase (ACC) catalyzes the rate-limiting step in fatty acid biosynthesis and autotrophic carbon fixation pathways across a wide range of organisms, making them attractive targets for drug discovery against various infections and diseases. Although structural studies on homomeric ACCs, which consist of a single protein with three subunits, have revealed the “swing domain model” where the biotin carboxyl carrier protein (BCCP) domain translocates between biotin carboxylase (BC) and carboxyltransferase (CT) active sites to facilitate the reaction, our understanding of the subunit composition and catalytic mechanism in heteromeric ACCs remains limited. Here, we identify a novel ACC from an ancient anoxygenic photosynthetic bacterium *Chloroflexus aurantiacus*, it evolves fused BC and BCCP domain, but separate CT components to form an enzymatically active ACC, which converts acetyl-CoA to malonyl-CoA *in vitro* and produces 3-hydroxypropionate (3-HP) via co-expression with recombinant malonyl-CoA reductase in *E. coli* cells. These findings expand the diversity and molecular evolution of heteromeric ACCs and provide a structural basis for potential applications in 3-HP biosynthesis.

## INTRODUCTION

Acetyl-CoA carboxylase (ACC) catalyzes the biotin-dependent carboxylation of acetyl-CoA (Ac-CoA) to malonyl-CoA (M-CoA). This reaction serves as the rate-limiting step in fatty acid biosynthesis of organisms ranging from bacteria to humans, as well as in the autotrophic carbon fixation pathways in extremophiles, such as *Metallosphaera sedula* (1, 2) and filamentous anoxygenic phototrophs (FAPs) (3–5). The diverse functions of ACCs make them attractive targets for drug discovery against microbial and fungal infections, type 2 diabetes, obesity, cancer, arteriosclerosis, and herbicides (6–8).

The carboxylation process involves three functionally distinct components. Using bicarbonate as the carboxyl donor, a biotin carboxylase (BC) catalyzes MgATP-dependent carboxylation of a biotin cofactor, which is covalently linked to a lysine residue within the highly conserved sequence motif (E-X-M-K-M) of a biotin carboxyl carrier protein (BCCP) (9). Subsequently, a carboxyltransferase (CT) facilitates the transfer of the carboxylate group from carboxylbiotin to Ac-CoA, resulting in the formation of M-CoA and regeneration of biotin-BCCP (10–12). Specifically, the BCCP biotinylation is catalyzed by biotinyl protein ligase [BPL, such as *E. coli* biotin inducible repressor (BirA) and mammal holocarboxylase synthase (HCS)] in an ATP-dependent manner (13–15). In eukaryotes, the BC, BCCP, and CT components can be integrated into a single polypeptide, forming a homomeric ACC. Alternatively, these components can also be separated or partially fused in different combinations to form an unstable heteromeric ACC in prokaryotes (16) and in the plastids of most plant cells, such as *Arabidopsis* and pea (17). A “swinging domain model” has been proposed for the carboxylation mechanisms of biotin-dependent carboxylases (18), in which the entire biotinylated BCCP domain translocates between the active sites of both BC and CT. This has been verified in the time-resolved cryo-electron microscopy (EM) studies of human pyruvate carboxylase (PC) (19). Enzymatic analyses revealed that the homomeric yeast ACC possesses higher activity (20) than that of the heteromeric *E. coli* ACC (21), indicating that fusion of these individual components is beneficial for increasing ACC activity.

BC catalyzes the initial first half-reaction of ACC, which involves the phosphorylation of bicarbonate by ATP to form a carboxyphosphate intermediate, followed by the transfer of the carboxyl group to biotinylated BCCP to form carboxybiotin. Structural studies have revealed a BC active site positioned between the ATP-binding and N/C-terminal sub-domains. When ATP binds, the ATP-binding sub-domain undergoes a rotation of approximately 45° to cover the active site (22). Within this active site, a strictly conserved glutamate residue facilitates the extraction of a proton from bicarbonate, enabling bicarbonate-initiated nucleophilic attack on ATP to form carboxyphosphate (10, 23). Although BC exists as a homodimer in solution, each subunit displays half-sites reactivity, whereby the two active sites alternate or ‘‘flip-flop’’ their catalytic cycles (24). In organisms such as *Streptomyces coelicolor* and *Mycobacterium tuberculosis*, the BC and BCCP are fused, called α subunit. However, the crystal structure of *M. tuberculosis* α-subunit (AccA3) only revealed a dimer of the ATP-bound BC domain, the BCCP was unresolved in the structure (25). Although the crystal structure of the recombinant *E. coli* BCCP-BC complex revealed a tetramer of two BC homodimers clamped by four BCCP molecules (16), the stoichiometry, structural, and functional correlations between the BC and BCCP subunits of the heteromeric ACCs remain controversial.

*Chloroflexus aurantiacus* is a representative bacterium of the FAPs, a diverse group of photosynthetic bacteria that perform anoxygenic photosynthesis and form the deepest branch of photosynthetic bacteria. Instead of the Calvin cycle that plants and algae use to assimilate carbon dioxide (26), FAPs, including *C. aurantiacus,* employ a 3-hydroxypropionate (3-HP) bi-cycle for autotrophic carbon fixation (3, 27). Within the consumption of five molecules of ATP and six molecules of NADPH, three molecules of bicarbonate are converted into one molecule of pyruvate through 19 reactions (3, 28, 29). The rate-limiting step of the 3-HP cycle is a heteromeric ACC-catalyzed conversion of Ac-CoA to M-CoA, which is further converted into 3-HP by a Malonyl-CoA reductase (MCR). Genomic analysis of *C. aurantiacu* has revealed the presence of genes encoding two BC isoforms, and separate BCCP, CTβ, and CTα subunits (4). The first BC isoform (WP_012257262, BC1) contains 596 amino acid residues, while the second BC isoform (WP_012259259, BC2) comprises 455 residues. During the transition from respiratory to phototrophic conditions, a simultaneous increase in the expression of *C. aurantiacus* BC1, CTβ, CTα, and most enzymes involved in the 3-HP cycle have been revealed in proteomic studies. By contrast, the expression level of BC2 gradually decreases during this transition (30). These findings suggest that BC1, but not BC2, is involved in the 3-HP carbon fixation pathway. However, the enzymatic activity of these two BC isoforms, as well as their interactions and coordination mechanisms with BCCP and CTβ-CTα subunits, remains unexplored. Further structural and functional investigations into this ACC could provide valuable insights into the catalytic mechanism and molecular evolution of heteromeric ACCs involved in autotrophic carbon fixation pathways.

Here, we identify a previously unrecognized *C. aurantiacus* BC1 that possesses fused BC and BCCP domains, enabling it to exhibit both biotin carboxylase and biotin carrier activities. Crystal structures of BC1 and BC2 at 3.2 Å and 3.0 Å resolutions, respectively, have revealed a BC1 tetramer consisting of two BC1-BC homodimers connected by an eight-stranded β-barrel of the BCCP domain. Removal of the BCCP and disruption of the β-barrel resulted in the dissociation of the tetramer, generating a dimeric structure resembling the BC2 homodimer resolved in both crystal structure and solution. The biotinylated BCCP domain further mediates the interactions between BC1 and CTβ-CTα, forming an enzymatically active heteromeric ACC, which converts Ac-CoA to M-CoA *in vitro*. Co-expression of this heteromeric ACC and MCR in *E. coli* BL21(DE3) cells facilitates 3-HP production, an important metabolic intermediate and platform chemical from biomass. This work identified a novel heteromeric ACC that evolves fused BC-BCCP domains to complete the ACC activity. The results of this study will broaden our understanding of the diversity and molecular evolution of heteromeric ACCs, and will provide the structural and functional basis for enzyme engineering and its applications in 3-HP biosynthesis.

## RESULTS

### *C. aurantiacus* BC1 possesses fused BC and BCCP domains

**Fig 1:**
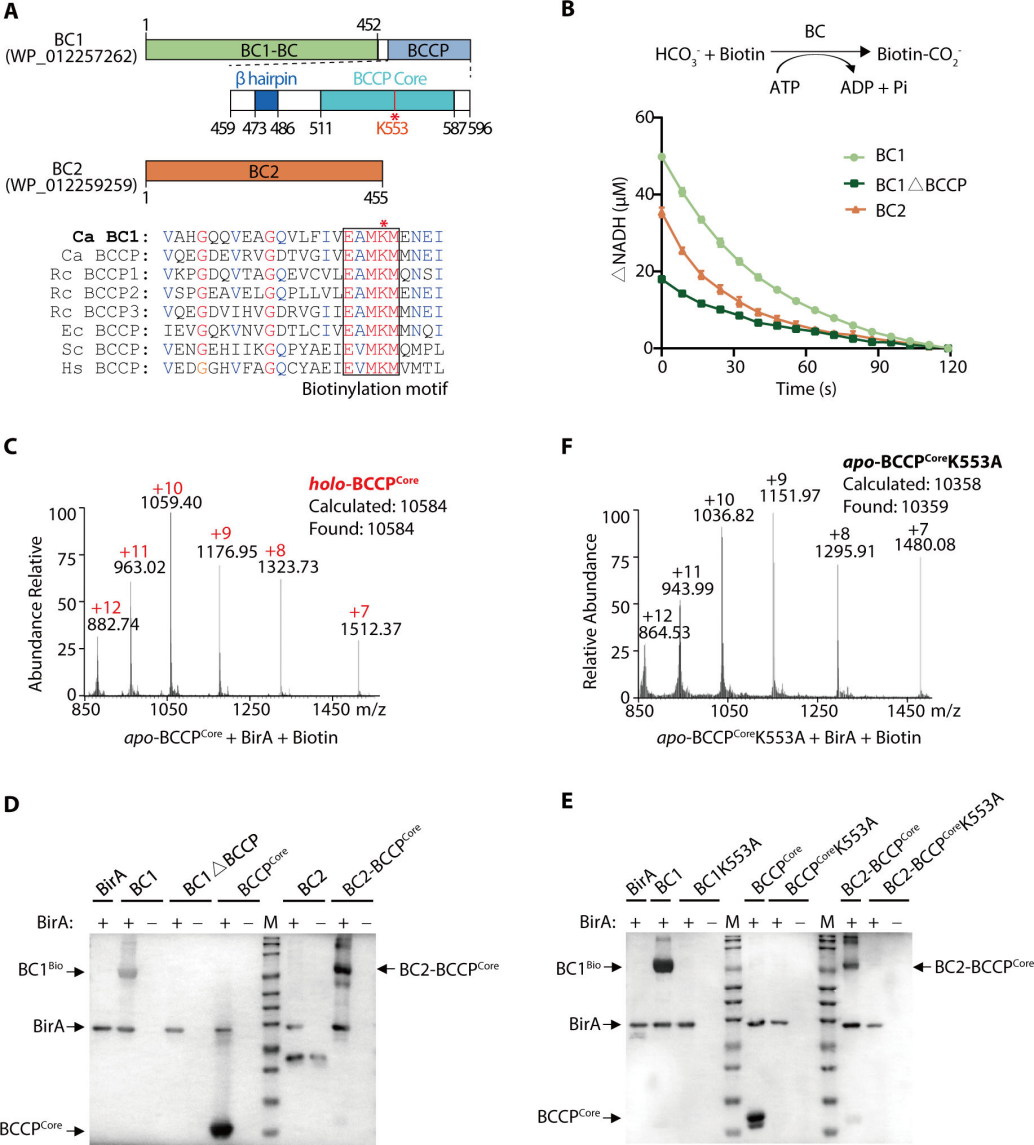
*Chloroflexus aurantiacus* BC1 is a bi-functional enzyme that confers fused BC and BCCP domains. (**A**) Diagram illustrating the primary structures of *C. aurantiacus* BC1 and BC2. *C. aurantiacus* possesses two BC isoforms, BC1 (WP_012257262) and BC2 (WP_012259259). BC1 confers a fused BC (Met1-Glu452) and BCCP domain (Ala459-Lys596), which contains a β hairpin (Thr473-Gly486) and a BCCP^Core^ (Lys511-Gln587) at the C-terminus. Multiple sequence alignment revealed the presence of a highly conserved biotinylation motif E-X-M-K-M (black box) in the BCCP^Core^. Ec, *Escherichia coli,* Ca, *Chloroflexus aurantiacus,* Rc, *Roseiflexus castenholzii,* Hs, *Homo sapiens,* Sc, *Saccharomyces cerevisiae*. The lysine residue (K553) is marked with a red star. (**B**) Biotin carboxylase activities of BC1, BC2, and the BCCP-truncated BC1 mutant (BC1ΔBCCP). Using 40 mM bicarbonate as substrate, and 40 mM biotin as carboxyl acceptor, the consumption of NADH was measured using 0.2 µM BC1 (or BC2, BC1ΔBCCP) as the enzyme, respectively. The absorption of NADH at 340 nm was plotted against the reaction time of 120 s. All data were obtained from three replicative experiments, with the mean and standard deviations calculated and plotted. (**C and F**) High-performance liquid chromatography-mass spectrometry (HPLC-MS) analyses to detect the biotinylation of the recombinant BCCP^Core^ domain. (**C**) Biotinylated *holo*-BCCP was produced when *apo*-BCCP was incubated with biotin and *C. aurantiacus* BirA. (**F**) When the K553A mutation was introduced, no biotinylated *holo*-BCCP was observed. The calculated and found molecular weights of *holo*-BCCP were both 10,584 Da. The calculated and found molecular weights of *apo*-BCCP were 10,358 and 10,359 Da, respectively. (**D and E**) Biotinylation of recombinant BC1, BC2, and mutant proteins by *C. aurantiacus* BirA was assessed. The biotinylated proteins were detected using HRP-conjugated streptavidin and visualized through HRP-DAB staining. The protein bands corresponding to the biotinylated BC1, BCCP, BC2-BCCP^Core^, and BirA were indicated with arrows. + and – represent the presence and absence of BirA in the reactions, respectively.

To explore the difference between *C. aurantiacus* BC1 and BC2 isoforms, we first reconstructed the neighbor-joining tree of BCs based on amino acid sequences of 24 BCs ranging from 15 kingdoms. The phylogenetic analyses indicated that *C. aurantiacus* BC1 and BC2 were originated from distinct *Chloroflexi* species (Fig. S1A). Multiple sequence alignment and conserved domain analyses revealed the presence of the biotin carboxylase (BC) domains in these BCs. Notably, *C. aurantiacus* BC1 but not BC2, as well as the BCs from *S. coelicolor*, *Roseiflexus castenholzii*, and *M. tuberculosis* possess a C-terminal biotin/lipoyl attachment domain (Fig. S1B). This domain often contains a conserved lysine residue that covalently binds the cofactor biotin or lipoic acid for mediating the carboxyl and acyl transfer reactions (31, 32). We further identified a consensus biotinylating motif (E^550^AMKM^554^) in the biotin/lipoyl attachment domains of *C. aurantiacus* BC1, and BCs from *S. coelicolor*, *R. castenholzii*, and *M. tuberculosis* (Fig. S1B). Coincidently, this motif is strictly conserved in *C. aurantiacus* BCCP, as well as *R. castenholzii*, *E. coli,* and eukaryotic BCCPs (Fig. 1A). In addition, BC1 contained 46.2% and 43.9% sequence identities with *M. tuberculosis* and *S. coelicolor* ACC α-subunit (Fig. S1B), which both contain a fused BC and BCCP domains for dual functionality (25). These observations suggested that *C. aurantiacus* BC1 most likely has a BCCP domain (Ala459-Lys596) fused to its BC domain (Met1-Glu452).

To verify whether *C. aurantiacus* BC1 confers both biotin carboxylase and biotin carrier activity, we expressed, purified, and verified the recombinant His_6_-tagged BC1, BC2 through high-performance liquid chromatography-electrospray tandem mass spectrometry (HPLC-ESI-MS/MS) (Fig. S2A through D). Using NaHCO_3_ and biotin as substrates, both BC1 and BC2 were enzymatically active in catalyzing the biotin carboxylation reactions (Fig. 1B). However, BC1 had a relatively higher substrate affinity and turnover number than BC2 (Table 1; Fig. S3A and B). Truncation of the BCCP domain (BC1ΔBCCP) substantially decreased but maintained the basal level BC activity (Fig. 1B; Fig. S3A and B; Table 1), indicating that the BCCP domain is dispensable for the biotin carboxylation activity of BC1. To test whether the BCCP domain can be biotinylated, we expressed and purified the individual C-terminal BCCP core (*apo*-BCCP^Core^) that contains the biotinylation motif (Fig. 1A). Then the *apo*-BCCP^Core^ was incubated with either *E. coli* or *C. aurantiacus* BirA in the presence of biotin and ATP. HPLC analyses revealed a mass increase of approximately 227 Da, which exactly matched the molecular weight of a single molecule of biotin (C_10_H_16_N_2_O_3_S) (Fig. 1C; Fig. S3C and D). These results indicated that the BC1-BCCP domain can be biotinylated by both *E. coli* and *C. aurantiacus* BirA.

**TABLE 1:** Kinetic parameters of *Chloroflexus aurantiacus* BC1, BC2, and BC1ΔBCCP when using NaHCO_3_ and biotin as the substrates

Protein	*K*_m_ (mM)^*a*^	*k*_cat_ (s^−1^)	*k*_cat_/*K*_m_ (mM^−1^ s^−1^)
With NaHCO_3_ as the varying substrate			
BC1	4.5 ± 0.1	20.7 ± 0.5	4.56 ± 0.09
BC1ΔBCCP	7.4 ± 0.3	14.3 ± 1.5	1.93 ± 0.08
BC2	6.7 ± 0.9	16.2 ± 0.1	2.38 ± 0.30
With biotin as the varying substrate			
BC1	11.4 ± 0.2	19.1 ± 1.1	1.67 ± 0.07
BC1ΔBCCP	17.1 ± 1.2	15.6 ± 0.3	0.92 ± 0.08
BC2	14.8 ± 2.2	16.7 ± 0.1	1.16 ± 0.16

^*a*^ The errors were obtained from fitting data to the Michaelis-Menten equation.

We then measured *C. aurantiacus* BirA-catalyzed biotinylation of the recombinant BC proteins (BC1, BC2, BC1ΔBCCP, and a chimera protein BC2-BCCP^Core^). The recombinant proteins were purified using streptavidin resin beforehand to remove the biotinylated fractions that were catalyzed by *E. coli* BirA during expression. For biotin labeling, each unbiotinylated protein (BC1, BC1ΔBCCP, BC2, BCCP^Core^, or BC2-BCCP^Core^) was incubated with *C. aurantiacus* BirA in the presence of biotin and ATP. The resultant biotinylated proteins were captured on Ni-NTA agarose to remove excess biotin, detected using horseradish peroxidase (HRP) conjugated streptavidin, and visualized by HRP-3, 3′-diaminobenzidine (DAB) staining. BC1, BCCP^Core^, and BC2-BCCP^Core^ can be biotinylated by BirA, but not BC1ΔBCCP and BC2 which lacked the BCCP domain (Fig. 1D; Fig. S3E). In particular, mutation of Lys553 in the conserved E^550^AMKM^554^ motif abolished the biotinylation activities of the BC1 and BCCP^Core^, as well as the chimera protein BC2-BCCP^Core^ (Fig. 1E; Fig. S3F). HPLC analyses confirmed that K553A mutation completely abolished the production of biotin-BCCP^Core^ (Fig. 1F). These results demonstrated that *C. aurantiacus* BC1 confers both biotin carboxylase and biotin carrier activities through its fused BC and BCCP domains, and Lys553 plays a critical role in BC1 biotinylation.

### The fused BCCP facilitates the formation of a BC1 tetramer

**Fig 2:**
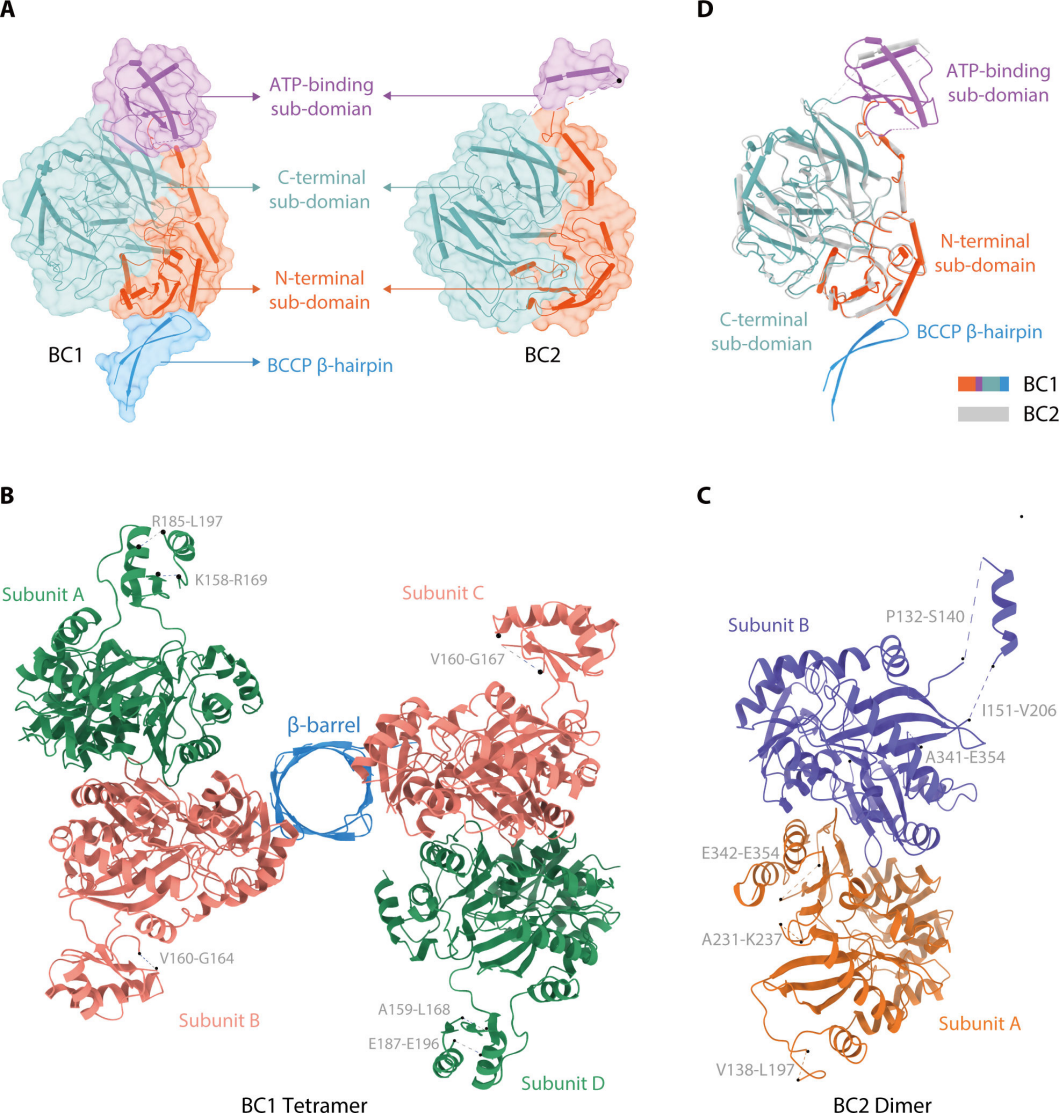
Crystal structures of *Chloroflexus aurantiacus* BC1 and BC2. (**A**) Overall structures of *C. aurantiacus* BC1 (left) and BC2 (right). The monomeric structures of BC1 and BC2 contain both N- (orange red) and C-terminal (cadet blue) sub-domains that form a Rossman fold, along with an ATP-binding sub-domain (medium orchid) extending away from these two sub-domains. BC1 features an additional BCCP domain (dodger blue), which was resolved with a β-hairpin containing two antiparallel β strands. (**B**) The BC1 tetramer is composed of two BC1-BC homodimers connected by a BCCP barrel at the N-terminal sub-domains. Different subunits are shown in distinct colors: sea green (subunits A and D) and salmon (subunits B and C). The BCCP domains are depicted in dodger blue. Gray dashed lines indicate amino acid residues with poor electron density. (**C**) Cartoon representation of BC2 homodimer. The two subunits are shown in chocolate and medium slate blue, respectively. Gray dashed lines indicate amino acid residues with poor electron density. (**D**) Superposition of the monomer structures of BC1 and BC2.

To investigate the structural basis of BC1 as a bi-functional enzyme, we determined the crystal structures of BC1 and BC2 at 3.2 Å and 3.0 Å resolutions, respectively (Fig. 2A; Table 2). Crystal packing analyses revealed two BC1 homodimers, but only one BC2 homodimer in each asymmetric unit (Fig. 2B and C). The superposition of one BC1-BC monomer with that of BC2 gave a root mean square deviation (RMSD) value of 0.654 Å (Fig. 2D), indicating that BC1 and BC2 possessed the same BC architectures. Each BC domain was composed of the N- and C-terminal sub-domains and an ATP binding sub-domain (Fig. 2A). It shared high sequence and structural conservations with the BCs from bacteria to human, especially the active site pocket (Fig. S4 and S5A ). Superposition of the BC1-BC domain with the *apo*- and *holo*-BCs from *E. coli*, *H. influenza*, *P. aeruginosa,* and yeast showed strict conservations at the BC domain, but the ATP binding sub-domain adopted a relative open conformation compared to that in the *holo*-BCs (Fig. S5B). These conserved structural features secured the biotin carboxylase activities of both BC1-BC and BC2.

**TABLE 2:** Crystal data collection and refinement statistics^*b*^

	*Chloroflexus aurantiacus* BC1 (PDB 8HZ4)	*Chloroflexus aurantiacus* BC2 (PDB 8HZ5)
Data collection		
Diffraction source	BL19U, SSRF	BL19U, SSRF
Wavelength (Å)	0.979	0.979
Space group	*P* 1 2_1_ 1	C 2 2 2_1_
Cell parameters (Å)	a = 128.528, b = 126.224, c = 132.192	a = 54.79, b = 154.092, c = 206.597
	α = γ = 90.0º, β = 106.437	α = β = γ = 90.0º
Total reflections	3150431	657622
Unique reflections^*a*^	66529 (6647)	17209 (1679)
R_merge_ (%)^*a*^	28.1(>100)	46.4 (>100)
I/σ (I)^*a*^	4.5 (0.857)	6.5 (1.5)
CC_1/2_^*a*^	0.895 (0.65)	0.884 (0.777)
Completeness (%)^*a*^	99.22 (99.67)	95.41 (95.45)
Refinement		
Resolution (Å)^*a*^	28.8–3.2 (3.314–3.2)	24.06–3.0 (3.107–3.0)
R_work_/R_free_ (%)	21.44 / 24.23	24.46 / 28.64
R.M.S. deviations		
Bonds (Å)	0.006	0.02
Angles (°)	0.84	1.84
Wilson B-factor	74.83	47.30
Average B-factor	70.85	37.22
Ramachandran plot		
Favored (%)	96.13	98.12
Allowed (%)	3.7	1.74
Outliers (%)	0.16	0.14

^*a*^ Statistics for the highest-resolution shell are shown in parentheses.

^*b*^ *R*_merge_ = ∑*_hkl_* ∑*_i_* │*I_i_(hkl)* -〈*I(hkl)*〉│/ ∑*_hkl_* ∑*_i_ I_i_(hkl)*, where *I_i_(hkl)* is the intensity of the *i*th measurement of reflection *hkl* and〈*I(hkl)*〉is the mean intensity of all symmetry-related reflections.

**Fig 3:**
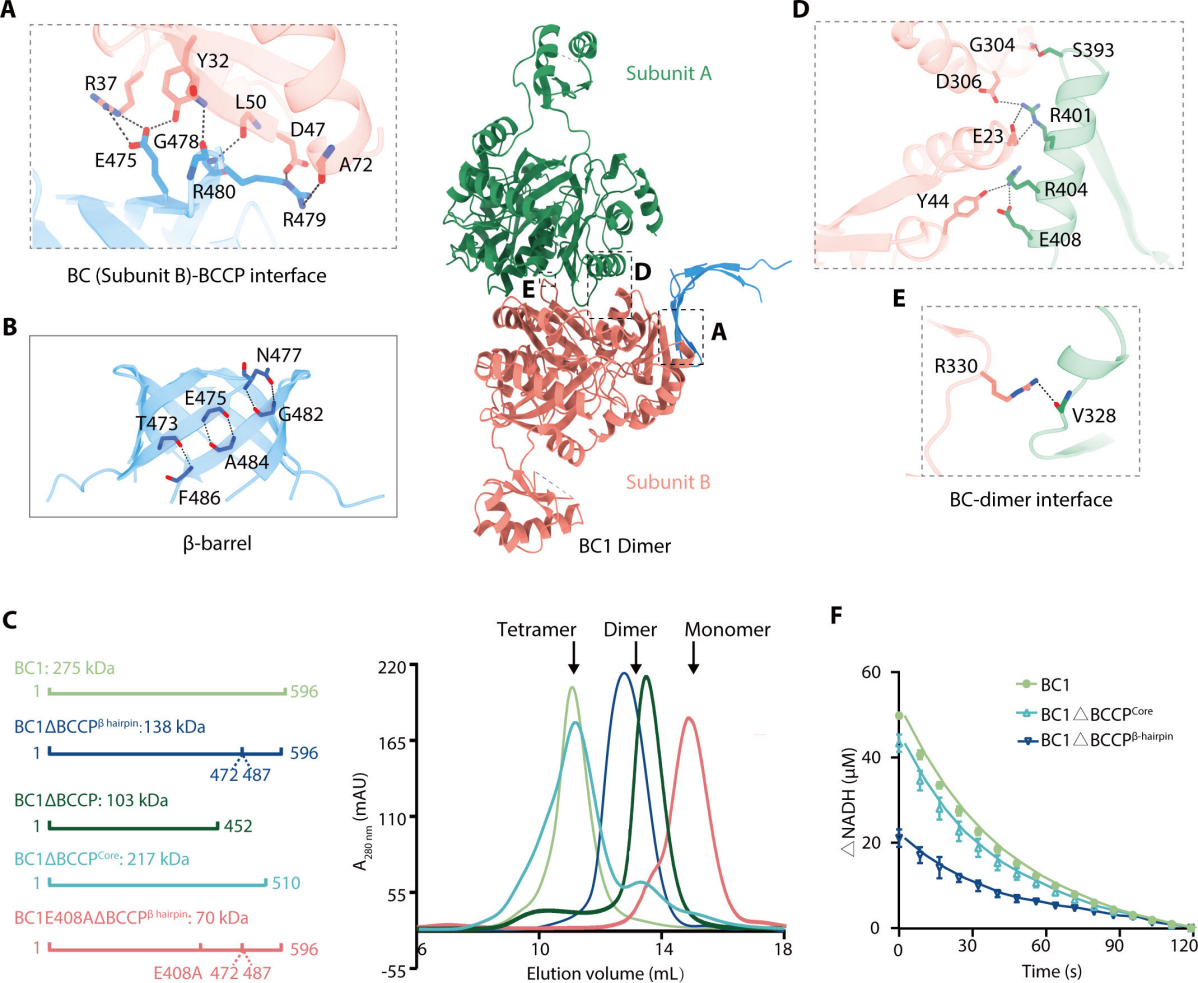
BCCP β-hairpin contributes to the tetramer formation and biotin carboxylase activity of *Chloroflexus aurantiacus* BC1. (**A**) Zoomed-in views highlight the BC-BCCP β-hairpin interface. Essential amino acid residues involved in mediating these interactions are displayed as stick models, and the hydrogen bonding interactions are indicated by dashed lines. (**B**) Amino acid residues (Thr473-Gly486) within the BCCP barrel form hydrogen bonding interactions. (**C**) Gel filtration and Sedimentation velocity AUC analyses of BC1 and its mutants. The diagram of the primary structures of BC1, and mutants BC1ΔBCCP, BC1ΔBCCP^Core^, BC1ΔBCCP^β-hairpin^, and BC1E408AΔBCCP^β-hairpin^ was shown on the left. The molecular weight detected by AUC was labeled. The gel filtration profile depicts the absorbance at 280 nm against elution volume (mL) from a HiLoad 10/300 Superdex 200 pg column. (**D and E**) Zoom in view of the BC1-BC dimer interface. The amino acid residues essential for mediating BC1-BC dimer interactions are shown in stick forms, and the hydrogen bonding interactions are indicated with dashed lines. (**F**) Biotin carboxylase activities of BC1, BC1ΔBCCP^Core^, and BC1ΔBCCP^β-hairpin^. The absorption of NADH at 340 nm was plotted against the reaction time of 120 s. All data were obtained from three replicative experiments, with the mean and standard deviations calculated and plotted.

Although we identified a complete BCCP domain (Ala459-Lys596) in *C. aurantiacus* BC1, the crystal structure only resolved an N-terminal β-hairpin (Pro466-Gly487) that composed of two antiparallel β-strands (Fig. 2A). This β-hairpin architecture has not been resolved in any previously reported biotin carboxylases structures. In particular, four β-hairpins cross-interlocked to form an eight-stranded β-barrel, which connected two BC1-BC homodimers at the N-terminal sub-domains to constitute a tetramer (Fig. 2B). The BC/β-barrel interface was mediated by a symmetrically distributed hydrogen bonding network, which was composed of amino acid residues Glu475, Gly478, Arg479, and Arg480 from the β-hairpin, Tyr32, Arg37, Asp47, Leu50 and Ala72 from the N-terminal sub-domain (Fig. 3A; Fig. S6A). Each β-hairpin was stabilized by hydrogen bonding interactions between Phe486-Thr473, Glu475-Ala484, and Asn477-Gly482 amino acid pairs (Fig. 3B). Consistent with the crystal structure, we also observed a BC1 tetramer in solution, through sedimentation velocity analytical ultracentrifugation (AUC) and gel filtration analyses (Fig. 3C; Fig. S7A). Specifically, truncation of the amino acid residues that constitute the entire BCCP domain (BC1ΔBCCP, Met1-Glu452) or the β-hairpin (BC1ΔBCCP^β-hairpin^, Thr473-Gly486) resulted in a dimer of the BC1 mutants (Fig. 3C; Fig. S7B and C). However, the tetramer formation of BC1 was not affected upon deletion of the BCCP core (BC1ΔBCCP^Core^, Met1-Lys510) (Fig. 3C; Fig. S7D). These results indicated that the β-hairpin but not the BCCP core mediated the formation of BC1 tetramer.

Each BC1-BC and BC2 homodimer was stabilized by extensive hydrogen bonding interactions between conserved amino acid pairs in the N- and C-terminal sub-domains (Fig. 3D and E;Fig. S6B and C). BC1-BC homodimer contains specific amino acid pairs Glu408-Tyr44, Ser393-Gly304, and Arg330-Val328 that are absent in BC2. Specifically, the dimer interface residues Tyr44, Val328, Ser393 in BC1-BC homodimer, and Arg310 in BC2 dimer are not conserved with other reported BCs (Fig. S4A). To differentiate the dimer formation of BC1ΔBCCP and BC1ΔBCCP^β-hairpin^ resulted from disruption of the BC1-BC dimer interface or the BC/β-barrel interaction, we mutated the dimer interface residues and measured the oligomerization state of these mutants. Gel filtration analyses both revealed that only the E408A mutation was capable of partially dissociating the BC1 tetramer (Fig. S6D). Incorporation of the E408A mutation into the mutant BC1ΔBCCP^β-hairpin^ (BC1E408AΔBCCP^β-hairpin^) further dissociated the dimer into a monomer (Fig. 3C; Fig. S7E), indicating that BC1ΔBCCP^β-hairpin^ dimer formation was indeed resulted from dissociation of the β-barrel. These observations confirmed the structural observations that the β-hairpin is critical for forming the BC1 tetramer (Fig. 2B). Furthermore, BC1 and BC1ΔBCCP^Core^ that existed as a tetramer in solution showed relatively faster NADH consumption rate than that of BC1ΔBCCP^β-hairpin^ (Fig. 3F). For BC2 that lacks a fused BCCP domain, it showed relatively lower biotin carboxylase activity and a dimer formation in solution (Fig. 1B; Fig. S6C and S7F). Overall, these results demonstrated that the β-hairpin mediated tetramer formation is necessary for maintaining effective biotin carboxylase activity of BC1.

### Biotinylated BC1 interacts with CTβ-CTα to form a detectable ACC complex

**Fig 4:**
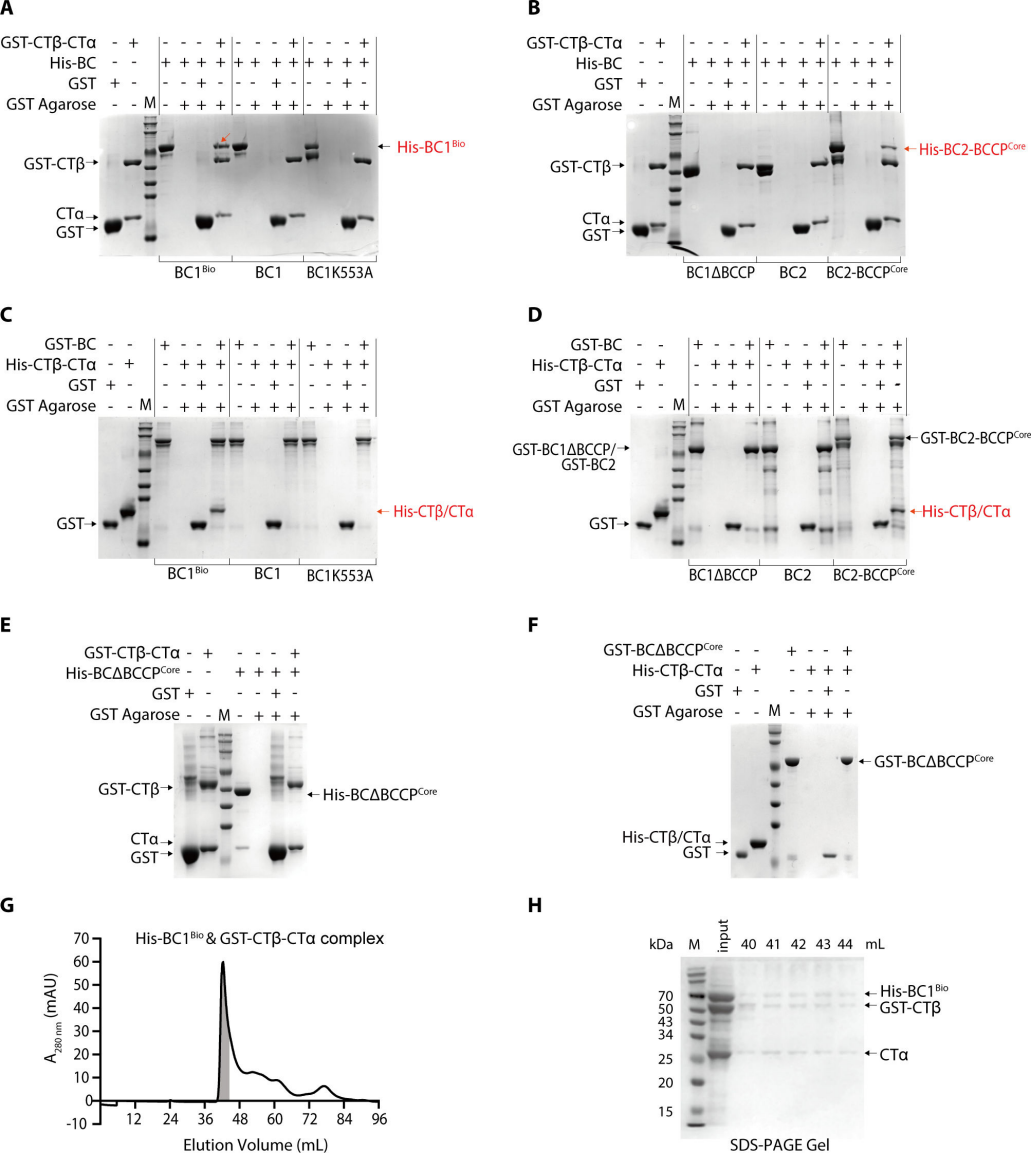
Biotinylated BCCP domain mediates direct interaction between *Chloroflexus aurantiacus* BC1 and CTβ-CTα. (**A–D**) Pull-down assays between GST-CTβ-CTα and His_6_-tagged biotinylated BC1, unbiotinylated BC1, BC1K553A (**A**), BC1ΔBCCP, BC2, and BC2-BCCP^Core^ (**B**). Similarly, assays using His-CTβ-CTα to pull down GST-tagged biotinylated BC1, unbiotinylated BC1, BC1K553A (**C**), BC1ΔBCCP, BC2, and BC2-BCCP^Core^ (**D**). The proteins were separated using 10% SDS-PAGE, and the protein marker ladders ranging from 250 to 10 kDa are shown. + indicates the addition of the corresponding proteins listed on the left. (**E**) Pull-down assays between GST-CTβ-CTα and His_6_-tagged BC1ΔBCCP^Core^. (**F**) Pull-down assays between His_6_ tagged CTβ-CTα and GST-BC1ΔBCCP^Core^. (**G**) Gel filtration analyses of the reconstructed ACC containing biotinylated His_6_-tagged BC1 & GST-CTβ-CTα. The gel filtration profile depicts the absorbance at 280 nm against elution volume (mL) from a HiLoad 16/600 Superdex 200 pg column. (**H**) SDS-PAGE analysis of the eluted peaks containing ACC complex from Fig. 3E (gray-colored fractions). The arrows denote the biotinylated His_6_-tagged BC1, GST-CTβ, and CTα.

Previous studies have demonstrated that the biotin-BCCP is essential for translocating the carboxybiotin intermediate between the BC and CT active sites (11, 16). To explore whether the biotinylated BCCP domain mediates the BC1 and CTβ-CTα interaction, we conducted pull-down assays toward the BirA-biotinylated BC1 and recombinant CTβ-CTα (Fig. S2E and F). Using unbiotinylated BC1 as a control group, GST-CTβ-CTα selectively retained the biotinylated BC1 (His-BC1^Bio^) and BC2-BCCP^Core^, but not BC2 or BC1ΔBCCP, BC1ΔBCCP^Core^ that lacked the BCCP domain (Fig. 4A, B and E). Conversely, the GST-tagged BC1^Bio^ and BC2-BCCP^Core^, but not BC2 or BC1ΔBCCP, BC1ΔBCCP^Core^, specifically captured His-CTβ-CTα (Fig. 4C, D and F). Consistently, the BC1K553A mutant showed no direct interaction with CTβ-CTα (Fig. 4A and C; Fig. S8A). These results confirmed that the biotinylation of BCCP is required for direct interactions between BC1 and CTβ-CTα. Notably, the interactions between BC1 and CTβ-CTα were not affected by the addition of substrates NaHCO_3_ and Ac-CoA facilitates in the pull-down analyses (Fig. S8B and C). To verify whether BC1^Bio^ and CTβ-CTα could form a complex *in vitro*, we co-transformed the constructs containing pET28a-*bc1* and pGEX-6p-1-*ctβ-rbs-ctα* into *E. coli* BL21(DE3) cells. Overexpression of these two plasmids and a two-step purification procedure yielded a single gel filtration peak that contains all three subunits of ACC (Fig. 4G and H). These analyses indicated that the biotinylated BC1 forms a detectable ACC complex with CTβ-CTα in solution.

### The ACC is enzymatically active *in vitro* and in the recombinant *E. coli* cells

**Fig 5:**
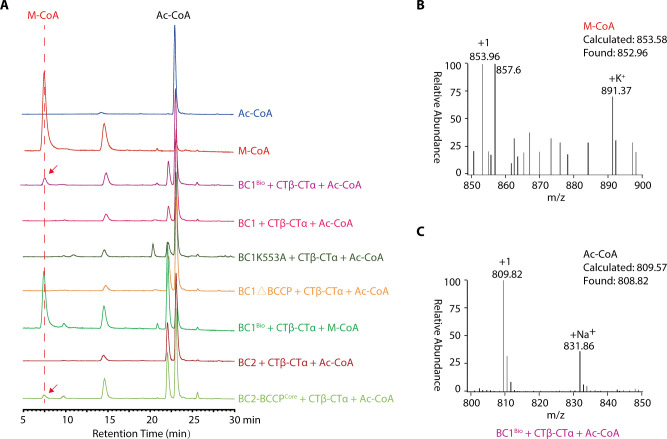
LC-MS analysis of *Chloroflexus aurantiacus* ACC catalyzed conversion of acetyl-CoA (Ac-CoA) to malonyl-CoA (M-CoA). (**A**) HPLC analysis of the reaction products catalyzed by biotinylated BC1 (BC1^Bio^, or unbiotinylated BC1, BC1K553A, BC1ΔBCCP, BC2, BC2-BCCP^Core^) in complex with CTβ-CTα. The absorbance of the chemicals at 260 nm (mAU) is plotted against the retention time. The peaks corresponding to Ac-CoA and M-CoA are detected at retention times of 22.5–23.5 min and 6–8 min, respectively. The production of M-CoA in the reactions was indicated with red arrows. (**B and C**) MS analyses of the reaction produced from reactions containing BC1^Bio^ + CTβ-CTα +Ac-CoA in Fig. 5A. The HPLC peaks at retention time 6–8 min (**B**) and 22.5–23.5 min (**C**) were detected, respectively. The calculated and found molecular weights of Ac-CoA and M-CoA are indicated.

To verify the activity of this ACC complex in catalyzing the biotin-dependent carboxylation of Ac-CoA, the biotinylated BC1 or BC2-BCCP^Core^ was each incubated with CTβ-CTα in the presence of substrates bicarbonate and Ac-CoA, and the cofactor ATP. HPLC analyses of the reaction products revealed the generation of a weak peak at a retention time of 6–8 min, which matched the retention time of the M-CoA standard sample (Fig. 5A). MS analyses further revealed a coincidence of the molecular weight of this peak with the M-CoA (Fig. 5B and C; Fig. S8D and E), indicating the production of M-CoA in these two reactions. However, no M-CoA production was detected in reactions containing the unbiotinylated BC1 (or BC1ΔBCCP, BC1K553A, BC2) (Fig. 5A). To investigate whether the low M-CoA yield resulted from the reverse reactions, M-CoA was used as a substrate to CTβ-CTα in the absence of Ac-CoA. As expected, a larger HPLC peak area corresponding to Ac-CoA appeared at the retention time of 22.5–23.5 min (Fig. 5A; Fig. S8D through G). These observations indicated that the reconstituted ACC complex is enzymatically active, and the occurrence of the reverse reaction limited M-CoA production.

**Fig 6:**
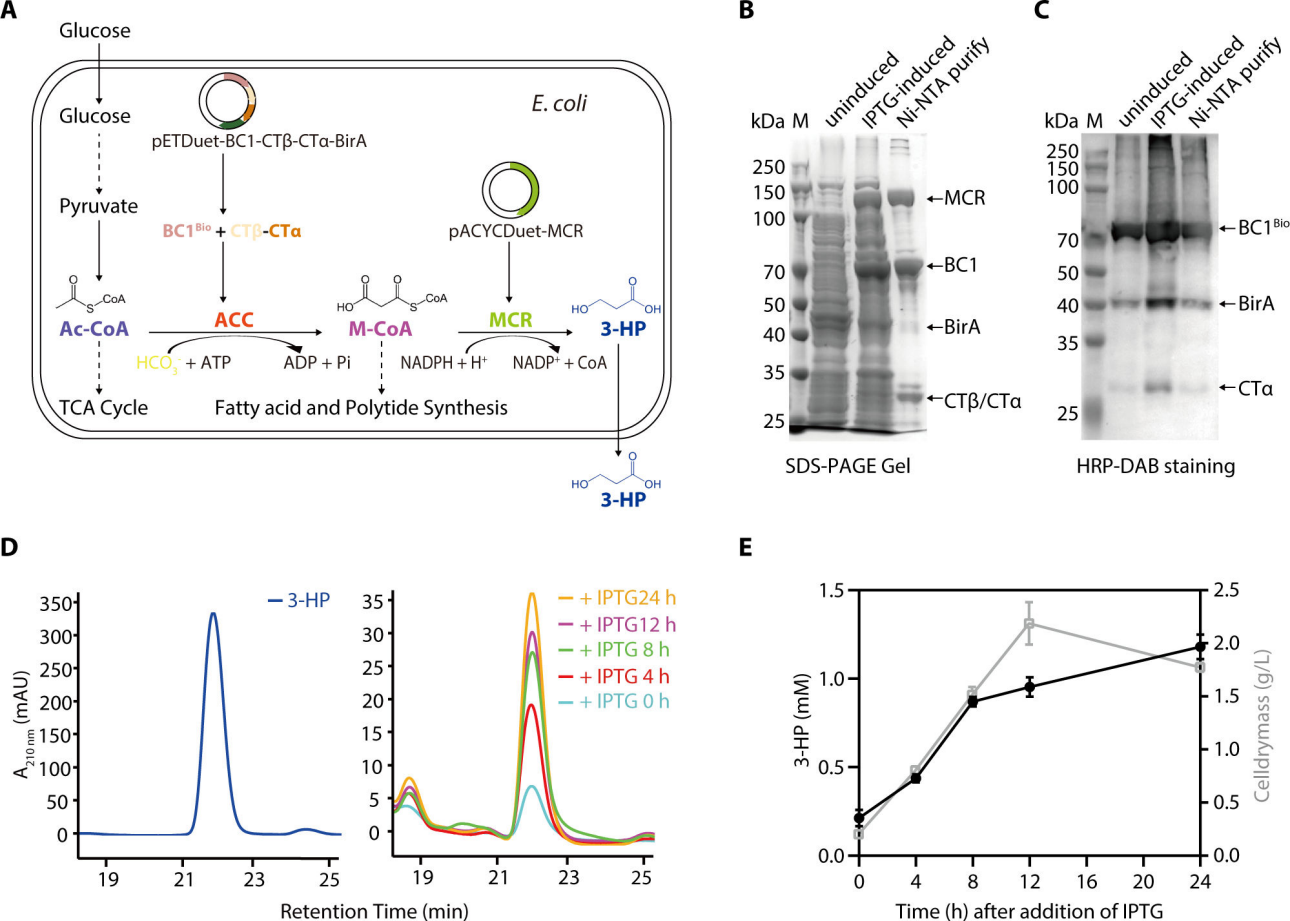
3-Hydroxypropionate (3-HP) production in *E. coli* BL21(DE3) cells that carry the reconstituted *Chloroflexus aurantiacus* ACC complex. (**A**) Scheme of the malonyl-CoA pathway in *E. coli*. Using glucose as the carbon source, pyruvate is converted to acetyl-CoA (Ac-CoA) by pyruvate dehydrogenase. Using bicarbonate as substrate, the Ac-CoA is converted to 3-HP through two consecutive carboxylation reactions involving co-expressed *C. aurantiacus* ACC and the bi-functional enzyme MCR that performs alcohol dehydrogenase and aldehyde dehydrogenase (CoA-acylating) activities in the N- and C-terminal fragments respectively. After induction, the co-expressed BirA catalyzes the biotinylation of BC1, which interacts with CTβ-CTα to form an active ACC complex. ACC catalyzes the conversion of Ac-CoA to M-CoA, which is further reduced by MCR to produce 3-HP. (**B**) SDS-PAGE analyses of the *E. coli* cell extracts and Ni-NTA purified fractions of the recombinant ACC and MCR. Arrows indicate the corresponding recombinant proteins. (**C**) HRP-DAB staining of Fig. 6B gel to show the biotinylated ACC subunits. (**D**) HPLC analysis of the 3-HP production in the recombinant cells cultured under aerobic conditions. The absorbance (mAU) of the chemicals at 210 nm is plotted against the retention time. The peaks corresponding to 3-HP are detected at retention times of 21–23 min. (**E**) Time-course profile of the cell mass (○) and 3-HP yield (●) are plotted against the culturing time. Data are shown as the mean ± standard deviations (*n* = 3).

*C. aurantiacus* ACC and MCR constitute a malonyl-CoA pathway, in which ACC-produced M-CoA is further converted to 3-HP by the bi-functional enzyme MCR (3, 29). To investigate whether consumption of M-CoA could facilitate the forward reaction of ACC, we constructed *E. coli* cells that carry the co-expressed *C. aurantiacus* BC1-CTβ-CTα, BirA, and MCR (Fig. 6A). SDS-PAGE analyses verified co-expression of these proteins in the cell extracts and Ni-NTA purified fractions (Fig. 6B), and the expression of the recombinant MCR was verified through HPLC-ESI-MS/MS (Fig. S2G and H). Streptavidin conjugated HRP-DAB staining showed clear bands of the biotinylated *C. aurantiacus* BC1, CTα, and BirA, indicating that the expressed ACC subunits can be biotinylated in the *E. coli* cells (Fig. 6C). To detect the 3-HP production yield, the *E. coli* cells carrying the reconstituted malonyl-CoA pathway were induced at 25°C in the presence of biotin and NaHCO_3_. HPLC analysis of the induced cells revealed a significant increase in the 3-HP yield, reaching 1.11 mM during a 24-h fermentation time (Fig. 6D and E). These results confirmed that the consumption of M-CoA by MCR facilitated the forward reaction of ACC in catalyzing the conversion of Ac-CoA. Overall, these analyses indicated that *C. aurantiacus* ACC containing BC1 evolved fused BCCP domain and CTβ-CTα is enzymatically active both *in vitro* and in the recombinant *E. coli* cells.

## DISCUSSION

ACCs are enzymes found in a wide range of organisms that play a crucial role in fatty acid metabolism, polyketide biosynthesis, and autotrophic carbon fixation pathways (29, 33, 34). Distinct from homomeric ACCs, heteromeric ACCs contain four functionally distinct components (BC, BCCP, CTβ, and CTα), which are either separated or partially fused in various combinations. However, an ACC with fused BC-BCCP and two separate CT subunits has not been reported. In this study, we identified a bi-functional BC isoform (BC1) from an ancient anoxygenic phototrophic bacterium *C. aurantiacus*. It confers both biotin carboxylase and biotin carrier activities with fused BC and BCCP domains. As a result, the biotinylated BC1 directly interacts with CTβ-CTα, forming a detectable ACC complex that catalyzes Ac-CoA carboxylation reactions *in vitro* and in the recombinant *E. coli* cells. In particular, we resolved the crystal structure of a previously uncovered BCCP β-hairpin. Four β-hairpins cross-interlocked into a β-barrel, which bridged two BC1-BC homodimers to form an enzymatically active tetramer. This is the first instance of a BCCP hairpin mediating the tetramer formation of BC.

Homomeric ACCs often have a BCCP domain fused to the BC via a BT or PT domain (19, 20, 35). Only a few heteromeric ACCs evolve with a fused BCCP component, such as the ones we identified in BCs from *C. aurantiacus*, *R. castenholzii*, *S. coelicolor*, and *M. tuberculosis* (Fig. S1A). Structural studies of both homomeric and heteromeric ACCs have revealed a consensus BCCP^Core^ structure, which contains four pairs of antiparallel β-sheets surrounding a hydrophobic core (11). Specifically, heteromeric BCCPs contain a widespread N-terminal extension composed of several β-hairpins, which are connected to the C-terminal BCCP^Core^ through random coils (Fig. S1; Fig. S9). However, no structural information is available for deciphering this specific extension in heteromeric BCCPs. Therefore, the resolved β-hairpin in our BC1 crystal structure gave a first glance into the N-terminal conformation of heteromeric BCCPs. Furthermore, we illustrated the essential role of the β-hairpin, but not the BCCP^Core^, in mediating the formation of an enzymatically active BC1 tetramer (Fig. 1B; Fig. S3A and B; Fig. 3F). By contrast, the *E. coli* BCCP-BC tetramer was stabilized by four BCCP^Core^ domains (16). Although homomeric ACCs contain a structured BT or PT domain (36), the BCCP β-hairpin does not show high sequence and structural similarity with these domains (Fig. S9). These differences indicated that the fused BCCP domain contributes unique structural and functional priorities to the BC component of heteromeric ACC (Fig. S6E).

To investigate the catalytic mechanism of *C. aurantiacus* BC1 that contains a fused BCCP, we first modeled biotin, cofactor Mg^2+^-ADP, and the substrate bicarbonate into the BC1-BC active site, based on the structure of substrate-bound *E.coli* BC (PDB 3G8C) (10–12), which shares 51.9% sequence identity and conserved architecture with BC1-BC (main chain RMSD of 0.735 Å). In the modeled BC1-BC active site, bicarbonate was immobilized through hydrogen bonds with the carboxyl oxygen of Glu295 (2.8 Å), which is conserved with Glu296 in *E. coli* BC that plays a role in stabilizing the bicarbonate during catalysis (Fig. S10A and B). The Mg^2+^-ADP and biotin were coordinated through hydrogen bonds with Glu275, Glu287, Glu200, Gln232 and Arg337, D382, respectively (Fig. S10B). These amino acid residues are strictly conserved in BCs from bacteria to humans (Fig. S4), indicating their same roles in catalyzing the biotin carboxylation reactions. Referring to the reported catalytic mechanism of BC (10–12, 23, 37), the strictly conserved Glu295 could act as a general base, extracting a proton from bicarbonate and initiating a nucleophilic attack on the γ-phosphate of ATP, leading to the formation of the carboxyphosphate. This unstable carboxyphosphate is then decomposed into CO_2_ and PO_4_^3-^. The PO_4_^3-^ could serve as the general base that extracts the proton from BCCP-conjugated biotin, leading to the enolization of the biotin ring. The biotin ureido anion then performs a nucleophilic attack on CO_2_ to form carboxybiotin.

BCCP plays an essential role in translocating the carboxyl group in both the biotin carboxylation and *trans*-carboxylation reactions of ACCs. To explore the structural basis of the fused BCCP domain in mediating biotin carboxylation, we simulated the full BC1 structural models by AlphaFold (38). Five BC1 models containing the complete BCCP domain were obtained with predicted local distance difference test (pLDDT) values above 87.3. Each model exhibited the BCCP domain adjacent to the BC1-BC domain (Fig. S10C). The simulated BCCP domain consisted of an N-terminal β-hairpin (Arg470-Vla485) connected to the C-terminal BCCP^Core^ (Ala520-Tyr586) through random coils. The BCCP^Core^ featured four pairs of antiparallel β-sheets surrounding a hydrophobic core, with the Lys553 residue in the β6-β7 hairpin oriented toward the BC1-BC active center (Fig. S10C). Comparing the modeled BC1 structure with the intermediate state of human PC (PDB 7WTE) revealed a considerable similarity in the BC and modeled BCCP^Core^ domains. In both structures, the amine sidechains of the biotinylated lysine residues were directed toward the BC1-BC active site (Fig. S10D). These findings suggest that the modeled BC1 conformation resembles the ATP-bound intermediate state of human PC.

Time-resolved cryo-EM structures of human PC have shown that ATP hydrolysis facilitates the translocation of BCCP from the CT active center to the BC active center (19, 35). Interestingly, when the modeled BC1-BC and BCCP domains were separately superposed on the ground state of human PC in the absence of ATP (PDB 7WTC), the BCCP^Core^ matched well with human PC BCCP domain but was flipped away from the BC1-BC active center (Fig. S10E). Similarly, in the crystal structure of the *E. coli* BCCP-BC complex, the BCCP domain was also directed outward from the BC active site, with the biotinylated Lys122 protruding away from the residue Glu295 at a distance of approximately 40 Å (16). Superposition of the BC1-BC crystal structure and the modeled BCCP domain with the *E. coli* BCCP-BC complex revealed conserved architectures at the BC and BCCP^Core^ (Fig. S10F). However, the N-terminal β-hairpins adopted dramatically different conformations (Fig. S10F), indicating significant structural flexibility in this region. The numerous random coils connected to the β-hairpin in *C. aurantiacus* BC1 probably provide sufficient structural flexibility for the fused BCCP domain to translocate between the ground state and intermediate state during biotin carboxylation reactions. In the ground state, the biotinylated Lys553 of the BCCP domain is positioned away from the active site of BC1-BC. Upon ATP binding, the BCCP domain could undergo conformational changes that translocate the BCCP^Core^ toward the BC1-BC active site, within which the biotinylated Lys553 is carboxylated (Fig. S10G).

After the biotin carboxylation reaction, CT facilitates the transfer of the carboxylate group from carboxylbiotin to Ac-CoA, resulting in the formation of M-CoA and regeneration of biotin-BCCP (10–12). In this study, we demonstrated that the biotinylated BCCP domain facilitates direct interactions between BC1 and CTβ-CTα, enabling the formation of a detectable ACC complex that catalyzes the conversion of Ac-CoA to M-CoA *in vitro* and in the recombinant *E. coli* cells (Fig. 5 and 6). However, we have not determined the structure of the *C. aurantiacus* CTβ-CTα subcomplex yet. Alternatively, CTα and CTβ contain high sequence identities with that of *E. coli* and *S. aureus* (41.2% and 43% for CTα, 42.6% and 40.3% for CTβ). Structural studies have revealed that *E. coli* and *S. aureus* CTα and CTβ both form heterotetramers (α_2_β_2_) to catalyze the *trans*-carboxylation reaction. The active site of each CTβ-CTα pair is located at the interface, with CTα-binding carboxybiotin and CTβ coordinating acetyl-CoA (39). However, the precise catalytic mechanism of heteromeric CTβ-CTα remains unclear, due to the lack of the BCCP-CT structures, and CT structures bound with acetyl-CoA and biotin. Further structural and functional investigations are required to elucidate the molecular mechanisms concerning BC1 and CTβ-CTα interactions.

In organisms that possess the fused BC and BCCP domains, such as *C. aurantiacus* and *R. castenholzii*, ACC plays a crucial role in catalyzing the rate-limiting step in the 3-HP autotrophic carbon fixation pathway (29). Remarkably, these two organisms both possess multiple BC isoforms(Fig. S1A). *R. castenholz*ii has three BC isoforms that share more than 41% sequence identity with *C. aurantiacus* BC1. Similar to *C. aurantiacus* BC2, *R. castenholz*ii BC1 does not contain a fused BCCP domain, while BC3 shares higher sequence identities (64.8%) with *C. aurantiacus* BC1. However, neither physiological roles nor catalytic mechanisms of these isoforms have been investigated yet. Here, we demonstrate that BC1, but not BC2, is capable of forming an enzymatically active ACC complex with CTβ-CTα, through biotinylation of the fused BCCP domain (Fig. 5 and 6). These results are consistent with previous *C. aurantiacus* proteomics time-course analyses, which showed a simultaneous increase in the expression levels of BC1 and CTβ, CTα (30). By contrast, the expression level of BC2 gradually decreases during the transition from respiratory to phototrophic conditions (30), suggesting that BC2 likely functions in pathways other than the 3-HP cycle. Since BC2 lacks the fused BCCP domain, it is probable that the BC2 participated biotin carboxylase reaction requires the involvement of a separate BCCP subunit (NCBI protein ID: ABY36917.1). The interaction network and catalytic mechanisms among these *C. aurantiacus* components, leading to the formation of a functional ACC, are currently being investigated in our laboratory.

In summary, we identify a previously unrecognized ACC from *C. aurantiacus*. It evolves fused BC and BCCP domain, but separate CT components to form an enzymatically active ACC, which converts Ac-CoA to M-CoA *in vitro* and produces 3-HP via co-expression with recombinant malonyl-CoA reductase in *E. coli* cells. The results of this study broaden our understanding of the diversity and molecular evolution of heteromeric ACCs and will lay a solid foundation for engineering the heteromeric ACCs and potential applications in 3-HP biosynthesis.

## MATERIALS AND METHODS

### Construction of the recombinant expression vectors

The gene sequences of *bc1* (*Caur*_1378), *bc2* (*Caur*_3421), *ctβ* (*Caur*_1647), *ctα* (*Caur*_1648), *bira* (*Caur*_0481), and *mcr* (*Caur*_2614) were amplified from *C. aurantiacus* J-10-fl genomic DNA. *E. coli bira* (NCBI Gene ID: 948469) was amplified from BL21(DE3) genomic DNA. Then the PCR product encoding the *C. aurantiacus* BC1 (Met1-Lys596) was inserted into the *Bam*H I and *Not* I sites of pET28a plasmid. The PCR product of BC1ΔBCCP (Met1-Glu452) was inserted into the *Bam*H I and *Xho* I sites of pET28a to construct the N-terminal His_6_-tagged expression vectors. The PCR products of *C. aurantiacus* BC2 (Met1-Val455), BCCP domain (Asp518-Lys596), BirA (Met1-Val283), MCR (Met1-Val1219), and *E. coli* BirA (NP_418404.1, Met1-K321) were inserted into the *Nde* I and *Xho* I sites of pET28a, respectively, to construct the N-terminal His_6_-tagged expression vectors. The BC2-BCCP^Core^ was an N-terminal His_6_-tagged chimera protein reconstructed using the homologous recombination method, in which the full-length BC2 (Met1-Val455) was fused with the BC1-BCCP domain (Asp518-Lys596). The gene sequences encoding CTβ (Pro24-Met305) and CTα (Met1-Asp253) were connected using an 18 bp sequence containing the *E. coli* ribosomal binding site (RBS, 5′-TATAAGAAGGAGATATAA-3′). Then the fused gene sequence was inserted into the pET28a vector for recombinant expression of an N-terminal His_6_-tagged CTβ-CTα subcomplex. Similarly, the expression vectors of the GST-tagged BC1, BC1ΔBCCP, BC2, BC2-BCCP^Core^, and CTβ-CTα were constructed using plasmid pGEX-6p-1 following the same procedure.

The expression vectors of pET28a-*bc1Δbccp^β-hairpin^* mutant of BC1 lacking the motif Thr473-Gly486 and pACYCDuet-*mcr* were constructed using the homologous recombination method. The expression vector encoding the BC1E408AΔBCCP^β-hairpin^ was amplified from the plasmid pET28a-*bc1Δbccp^β-hairpin^* by PCR using a QuickChang site-directed mutagenesis kit (Strata-gene, Santa Clara, CA, United States). The synthetic operon was constructed by sequentially cloning *bc1*, *ctβ,* and *ctα* genes in the MCS-I of the pETDuet vector under the control of the T7 promoter. The RBS (5′-TATAAGAAGGAGATATAA-3′) was incorporated at the upstream region of the start codon of each gene, except for *bc1*, in which vector *rbs* was utilized. The *C. aurantiacus birA* was amplified as a single gene fragment and ligated into the MCS-II of the pETDuet vector using the homologous recombination method. All the constructed vectors and gene sequences were confirmed by DNA sequencing.

### Protein expression and purification

Each sequenced plasmid was transformed into *Escherichia coli* BL21(DE3) cells for recombinant expression of the N-terminal His_6_-tagged or GST-tagged BC1, BC1ΔBCCP, BCCP, BC1ΔBCCP^β-hairpin^, BC1E408AΔBCCP^β-hairpin^, BC2, BC2-BCCP^Core^, CTβ-CTα, BirA of *C. aurantiacus*, *E. coli* BirA, and BC1-CTβ-CTα-BirA-MCR, respectively. The transformed cells were grown in 1 L Luria-Bertani broth containing 50 µg mL^−1^ kanamycin for the cells containing pET28a expression vectors, 100 µg mL^−1^ ampicillin for cells containing pGEX-6p-1 plasmids, 100 µg mL^−1^ ampicillin and 34 µg mL^−1^ chloramphenicol for cells containing pETDuet and pACYCDuet plasmids at 37°C until the OD_600_ reached 0.6–0.8. The gene expression of each recombinant protein was then induced with isopropyl-β-D-thiogalactopyranoside (IPTG) overnight at 16°C.

Cells were harvested by centrifugation at 7,500 × *g* for 10 min at 4°C and were resuspended in corresponding buffers prior to homogenization with a high-pressure homogenizer (Union, People’s Republic of China). For His_6_-tagged recombinant proteins, the lysis buffer contained 20 mM Tris-HCl pH 8.0, 150 mM NaCl, and 5 mM MgCl_2_. The cells expressing His_6_-CTβ-CTα were lysed with buffer containing 50 mM Tris-Base pH 9.0, 300 mM NaCl, and 5% glycerol. The cells that co-expresses His_6_-BC1-CTβ-CTα-BirA and His_6_-MCR were lysed by 50 mM Tris-Base pH 9.0, 150 mM NaCl, 5 mM MgCl_2_, and 5% glycerol. The insoluble cell debris was removed by centrifugation at 22,000 × *g* for 40 min at 4°C. The supernatant containing crude soluble proteins was loaded onto a Ni^2+^-chelating affinity chromatography column (GE Healthcare, Cytiva, USA) and was rinsed with lysis buffer containing 10 mM imidazole to remove non-specifically bound proteins. The bound recombinant proteins were eluted with the lysis buffer containing 250–300 mM imidazole. The elutes were further purified by a HiLoad 16/600 Superdex 200 PG size exclusion column (GE Healthcare, Cytiva, USA) to 95% purity. The gel filtration buffer for purifying the His_6_-tagged recombinant proteins contained 20 mM Tris-HCl pH 8.0, 150 mM NaCl, and 5 mM MgCl_2_. Alternatively, gel filtration of His_6_-CTβ-CTα was performed using 50 mM Tris-Base pH 9.0, 300 mM NaCl, and 5% glycerol. The gel filtration buffer for His_6_-BC1-CTβ-CTα-BirA-MCR contains 50 mM Tris-Base pH 9.0, 150 mM NaCl, 5 mM MgCl_2_, and 5% glycerol, respectively.

The GST-tagged recombinant proteins were purified using Glutathione sepharose Sepharose 4B agarose (GE Healthcare, Cytiva, USA). After centrifugation, the supernatant containing crude soluble proteins was loaded onto a GST affinity chromatography column and was rinsed with phosphate-buffered saline buffer to remove non-specifically bound proteins. The bound recombinant proteins were eluted with the elution buffer (20 mM Tris-HCl pH 8.0, 150 mM NaCl, 5 mM MgCl_2_ for BC1, BC1ΔBCCP, BC2, and BC2-BCCP^Core^; 50 mM Tris-Base pH 9.0, 300 mM NaCl and 5% glycerol for CTβ-CTα) containing 20 mM reduced glutathione. The eluent was further purified by a HiLoad 16/600 Superdex 200 PG size exclusion column (GE Healthcare) to 95% purity.

To remove the endogenous biotinylated proteins, the GST- and His_6_-tagged proteins were incubated with streptavidin agarose resin for 1 h at 4°C. Then the endogenously biotinylated proteins were eluted with 2.5 mM *d*-Desthiobiotin, and the elutes that contained the non-biotinylated proteins were collected and dialyzed against buffer containing 20 mM Tris-HCl pH 8.0, 150 mM NaCl, 5 mM MgCl_2_, 0.5 mM DTT for BC1, BC1ΔBCCP, BC2, BC2-BCCP^Core^, and 50 mM Tris-Base pH 9.0, 300 mM NaCl, 5% glycerol, 5 mM MgCl_2_, and 0.5 mM DTT for the CTβ-CTα.

### Enzymatic analysis of the biotin carboxylase activity

Biotin carboxylase activity was determined spectrophotometrically by measuring the ATP hydrolysis rate as described (40). The BC-catalyzed ADP production was coupled to pyruvate kinase (PK) that transfers a phosphate group from phosphoenolpyruvate (PEP) to ADP for pyruvate generation, and lactate dehydrogenase (LDH) that catalyzes the NADH-dependent conversion of pyruvate to lactate. The absorbance of NADH at 340 nm was recorded for 4 min at 25°C to detect the biotin carboxylase activities. The standard assay mixture (100 µL) contains 100 mM Hepes pH 8.0, 2 mM ATP, 8 mM MgCl_2_, 40 mM biotin, 40 mM bicarbonate, 0.2 mM NADH, 0.5 mM PEP (Aladdin, China), 6.5 units of LDH (Yuanye, China), 3.4 units of PK (Yuanye, China), and 0.2 µM BC1 (BC1ΔBCCP^Core^, BC1ΔBCCP^β-hairpin^, BC1ΔBCCP or BC2). Enzyme concentration was determined by the Bradford method. The apparent Michaelis-Menten constant (*K_m_*) and V*_m_* of bicarbonate and biotin were measured with a standard reaction mixture containing varied concentrations of bicarbonate (2.5, 5, 7.5, 10, 15, 20, 40, 60, and 80 mM) and biotin (2.5, 5, 7.5, 10, 15, 20, 40, 60, and 80 mM), respectively. Linear initial rates of bicarbonate (or biotin) at different concentrations were fitted using the Michaelis-Menten model in Prism8. All the enzymatic data were obtained from triplicate experiments, with the mean and standard deviations calculated and plotted.

### Measurements of the biotin carboxyl carrier activity

The biotin carboxyl carrier activity of BC1, BC2, and the mutants was measured by detecting the BirA-catalyzed biotinylation of the purified recombinant proteins. The recombinant proteins were first purified using streptavidin resin, which removed the biotinylated fractions that were catalyzed by *E. coli* BirA during expression. Reaction conditions were adapted as previously described (41). For each reaction, 2.5 µM of the un-biotinylated BC1 (or BCCP, BC1ΔBCCP, BC2, BC2-BCCP^Core^) was incubated with the assay solution containing 100  mM Tris-HCl pH 8.0, 150   mM NaCl, 5 mM MgCl_2_, 0.5 mM DTT, 10 µM biotin, 1 µM *C*. *aurantiacus* BirA (or *E. coli* BirA), 0.3 mM ATP for 1 h at 37°C. The reaction mixture in the absence of BirA was used as the control group. After incubation, samples were incubated with 10 µL Ni-NTA agarose at 4°C for 10 min. The supernatant from centrifugation was discarded to remove excess biotin, and the Ni-NTA agarose was washed three times with 1 mL buffer containing 50 mM Tris-HCl pH 8.0, 150 mM NaCl, and 5 mM MgCl_2_. The biotinylated protein samples were then eluted from the Ni-NTA agarose using 30 µL of 250 mM imidazole in 50  mM Tris-HCl, pH 8.0.

The resultant biotinylated proteins were detected by HRP-conjugated streptavidin and visualized by HRP-DAB staining (Beyotime, China). The captured protein samples were separated on an SDS-PAGE gel and transferred to a PVDF membrane in transfer buffer (25 mM glycine, 50 mM Tris, 20% methanol) by a *Trans* Turbo Blot system (Biorad, American). The PVDF membrane was blocked with TBST buffer (150 mM NaCl, 10 mM Tris-HCl pH 7.5 with 0.1% Tween 20) and 0.05 g mL^−1^ skimmed milk for 1 h at room temperature (RT), then was western blotted using 1:2,000 dilution of HRP-Streptavidin (Beyotime, China). Then each of the membranes was washed five times with TBST and visualized by HRP-DAB staining (Beyotime, China).

### HPLC-MS analysis of the biotin-transfer and acetyl-CoA carboxylation reactions

The reaction mixtures for measuring the BCCP activity were further analyzed by HPLC-MS, which was equipped with an Agilent 1200 HPLC system (Agilent, Santa Clara, CA. USA) and a Thermo Finnigan LCQDeca XP Max LC/MS system (Thermo Finnigan, Waltham, MA, USA). The samples were separated on an Agilent SB-C18 column (3.5 µm particle size, 80 Å, 2.1 × 150 nm), using 0.1% formic acid as solvent A and acetonitrile as solvent B at 35°C. The following binary gradient was used for elution: a 90%–70% linear gradient of solvent A for 0–5 min, a 70%–50% linear gradient of solvent A from 5 to 55 min, a linear gradient from 50% to 30% solvent A from 55 to 60 min, and equilibration to initial conditions for 13 min at a flow rate of 0.2 mL.min^−1^. UV detection was performed at both 220 and 280 nm. MS with an electrospray ionization (ESI) source was performed as follows: positive mode, source voltage of 2.5 kV, capillary voltage of 41 V, sheath gas flow of 45 arbitrary units, auxiliary/sweep gas flow of 5 arbitrary units, capillary temperature 330°C.

The production of M-CoA was detected by HPLC. In the presence of 10 mM Ac-CoA, 40 mM bicarbonate, and 1 mM ATP, 5 µM biotinylated BC1 (or BC1ΔBCCP, BC2, BC2-BCCP^Core^) was incubated with 5 µM purified CTβ-CTα in buffer containing 50 mM Tris-Base pH 9.0, 300 mM NaCl, 5 mM MgCl_2_, and 0.5 mM DTT for 1.5 h at RT. The reaction mixtures were monitored by HPLC using an Agilent Extend-C18 column (5 µm particle size, 80 Å, 4.6 × 250 nm). The separation was performed with 10 mM ammonium acetate as solvent A, and methyl alcohol as solvent B at 35°C. The following binary gradient was used: equilibration at 2% solvent A for 10 min, a linear gradient from 2% to 5% solvent A from 10 to 15 min, a linear gradient from 5% to 20% solvent A from 15 to 30 min, a linear gradient from 20% to 80% solvent A from 30 to 35 min, and equilibration to initial conditions for 15 min. The flow rate is 1 mL.min^−1^. UV detection was performed at 260 nm.

### Crystallization of *C. aurantiacus* BC1 and BC2

The purified *C. aurantiacus* BC1 and BC2 were concentrated using an Amicon Ultra centrifugal filter device (10 kDa molecular weight cutoff, Millipore) at 4°C. Protein concentrations were determined using a Nanodrop device (IMPLEN) by recording the absorption at 280 nm. The protein samples were diluted to 6.5 mg·mL^−1^ for BC1 and 10 mg mL^−1^ for BC2 in buffer (20 mM Tris-HCl pH 8.0, 150 mM NaCl, 2 mM MgCl_2_) for crystallization. Crystallization was performed using the hanging-drop vapor diffusion method, with 1.2 µL of protein sample mixed with an equal volume of reservoir solution, and the mixture was equilibrated against 200 µL reservoir solution. Crystals of bicarbonate bound BC1 were obtained in the reservoir solution containing 0.1 M Bicine pH 9.0, 2% (vol/vol) 1,4-Dioxane, 10% (wt/vol) PEG20,000, and 5% (wt/vol) PEG550 at 16°C. The bicarbonate bound BC2 was crystallized with a reservoir solution containing 28% (vol/vol) isopropyl alcohol, 0.1 M Bis-Tris pH 6.5, 4% (vol/v) PEG200 at 16°C.

### Crystal diffraction data collection, structure determination, and refinement

The optimized crystals were cryo-protected by adding 30% glycerol to the reservoir solution and flash-freezing with liquid nitrogen. A 3. 2 Å data set of BC1 and a 3.0 Å data set of BC2 were both collected at SSRF BL19U (Table 2). Diffraction data were automatically processed, integrated, and scaled with Porpoise XDS software (42). The quality of the data was assessed using SFCHECK (43), and the solvent content was calculated using Matthews_Coef from the CCP4 package (44). The BC1 and BC2 structures were determined by molecular replacement method, using the structures of *H. influenza* BC (PDB 4MV1) (45) and *P. aeruginosa* BC (PDB 2C00) (24) as the search model for BC1 and BC2, respectively. The Phaser program (46) from the CCP4 package was employed to determine the initial phases; iterative model building and refinement were performed using Coot (47), Refmac5 (48) and Phenix (49) to obtain the refined model (Table 2).

### Sedimentation velocity analytical ultracentrifugation

AUC was performed to check the oligomerization states of BC1, BC1ΔBCCP, BC1ΔBCCP^β-hairpin^ BC1ΔBCCP^Core^, BC1E408AΔBCCP^β-hairpin^, and BC2 in solution. Sedimentation experiments were performed on a Beckman Coulter Proteome Lab XL-I ultracentrifuge using a 4-hole An-60Ti rotor. Protein samples with an initial absorbance at 280 nm of ~0.7 were equilibrated for 2 h at 20°C under a vacuum prior to sedimentation. The absorbance at 280 nm was measured using a continuous scan mode during sedimentation at 42,000 rpm in 12 mm double-sector cells. The data were analyzed using Sedfit (50).

### Pull-down assays

For pull-down assays that use GST-tagged CTβ-CTα as bait, 50 µL of glutathione-Sepharose resin was incubated with 50 ng GST-tagged CTβ-CTα in 500 µL binding buffer (50 mM Tris-Base pH 9.0, 150 mM NaCl, 5 mM MgCl_2_, 5% glycerol, and 0.01% Triton-X100). After three times washing of the resin, 150 ng of biotinylated His_6_-BC1 (or unbiotinylated His_6_-BC1, His_6_-BC1K553A, His_6_-BC1ΔBCCP, His_6_-BC2, His_6_-BC2-BCCP^Core^) was incubated with the resin for 1 h at 4°C. After five times washing with binding buffer, the resin was resuspended in 30 µL SDS-PAGE loading buffer, denatured at 100°C for 10 min, and then was detected by SDS-PAGE. The pull-down assays targeting the His-tagged CTβ-CTα by GST-fused BC1 (or BC1ΔBCCP, BC2, BC2-BCCP^Core^) were performed following a similar procedure. To test whether the addition of substrates NaHCO_3_ and Ac-CoA facilitates BC1 and CTβ-CTα interactions, the steps of the pull-down experiments were the same as those mentioned above, except that 2 mM NaHCO_3_ and Ac-COA were added to the incubated solutions when His_6_-BC1 (or His_6_-BC2, their mutants) was added.

To obtain the protein complex of His_6_-BC1 and GST-CTβ-CTα, the *E. coli* BL21(DE3) cells carring the plasmids pET28a-*bc1* and pGEX-6p-1-*ctβ-rbs-ctα* were grown in 1 L Luria-Bertani medium containing 50 µg mL^−1^ kanamycin and 100 µg mL^−1^ ampicillin at 37°C until the OD_600_ reached 0.6–0.8. Then the recombinant protein expression was induced by the addition of 0.3 mM IPTG and 10 mg/L biotin overnight at 16°C. The cells were lysed in a buffer containing 50 mM Tris-Base pH 9.0, 150 mM NaCl, 5 mM MgCl_2_, and 5% glycerol. The His_6_-BC1 and GST-CTβ-CTα complex was obtained by two-step purification involving Ni^2+^-chelating and GST affinity chromatography. The elutes containing the His_6_-BC1 and GST-CTβ-CTα complex were further purified by a HiLoad 16/600 Superdex 200 PG size exclusion column (GE Healthcare, Cytiva, USA).

### 3-HP production in the recombinant *E. coli* cells

The plasmids pETDuet-*bc1-ctβ-ctα-birA* and pACYCDuet-*mcr* were transformed into *E. coli* BL21 (DE3) cells. The cells were cultivated in M9 medium (0.2 g yeast extract, 1.0 g NH_4_Cl, 1.0 g NaCl, and 0.25 g MgSO_4_·7H_2_O. 20 g glucose dissolved in per liter deionized water) that was supplemented with 0.1 M potassium phosphate buffer, 100 µg mL^−1^ ampicillin, and 34 µg mL^−1^ chloramphenicol. The recombinant cells were cultured aerobically in 250 mL screw-capped Erlenmeyer flasks containing 10 mL of M9 medium at 37°C until OD_600_ reaches ~0.6. The cells were then induced with 50 µM IPTG at 25°C, along with the addition of 40 mg/L biotin and 20 mM NaHCO_3_. Starting at the induction time, the medium at time points (0, 4, 8, 12, and 24 h) were collected and centrifugated at 10,000 × *g* for 10 min. Then the supernatant at each time point was filtered through a Tuffryn membrance (Acrodisc, Pall Life Sciences) and eluted using 2.5 mM H_2_SO_4_ through a 300 × 7.8 mm Aminex HPX-87H (Bio-Rad, USA) column at 65°C with 0.5 mL min^−1^ flow rate. The 3-HP yield was then detected by HPLC at a wavelength of 210 nm. The sediments at each time point were dried to detect the dry weight of the cells. The 3-HP yield and the cell mass were obtained from triplicate experiments, with the mean and standard deviations calculated and plotted.

## Data Availability

The structure factors and coordinates of BC1 and BC2 have been deposited in the Protein Data Bank under the accession codes 8HZ4 and 8HZ5. Other data are available from the corresponding author upon reasonable request.

## SUPPLEMENTAL MATERIAL

Supplemental figures mbio.03414-23-s0001.docx Figures S1-S10.

ASM does not own the copyrights to Supplemental Material that may be linked to, or accessed through, an article. The authors have granted ASM a non-exclusive, world-wide license to publish the Supplemental Material files. Please contact the corresponding author directly for reuse.
