# Supplementary material for: Chloroflexus aurantiacus acetyl-CoA carboxylase evolves fused biotin carboxylase and biotin carboxyl carrier protein to complete carboxylation activity
Source: mBio. 2024 Apr 4;15(5):e03414-23. doi: 10.1128/mbio.03414-23 (PMC11077971; doi:10.1128/mbio.03414-23)
Supplement: Supplemental figures — Figures S1-S10. [file mbio.03414-23-s0001.docx]

**Supplemental Materials for**

***Chloroflexus aurantiacus* acetyl-CoA carboxylase evolves fused biotin carboxylase and biotin carboxyl carrier protein to complete carboxylation activity**

Jiejie Shen^1,2,#^, Wenping Wu^1,2,#^, Kangle Wang^1,2^, Jingyi Wu^1,2^, Bing Liu^1,2^, Chunyang Li^1,2^, Zijun Gong^1,2^, Xin Hong^1,2^, Han Fang^1,2^, Xingwei Zhang^3^, Xiaoling Xu^1,2,4,^*

^1^Department of Biochemistry and Molecular Biology, School of Basic Medical Sciences, Hangzhou Normal University, Hangzhou, 311121, China

^2^Zhejiang Key Laboratory of Medical Epigenetics, Hangzhou Normal University, Hangzhou, 311121, China

^3^The Affiliated Hospital of Hangzhou Normal University, Hangzhou Normal University, Hangzhou, 311121, China

^4^Photosynthesis Research Center, College of Life and Environmental Sciences, Hangzhou Normal University, Hangzhou, 311121, China

^#^These authors contributed equally to this work.

*Correspondence: Xiaoling Xu (xuxl@hznu.edu.cn)

**
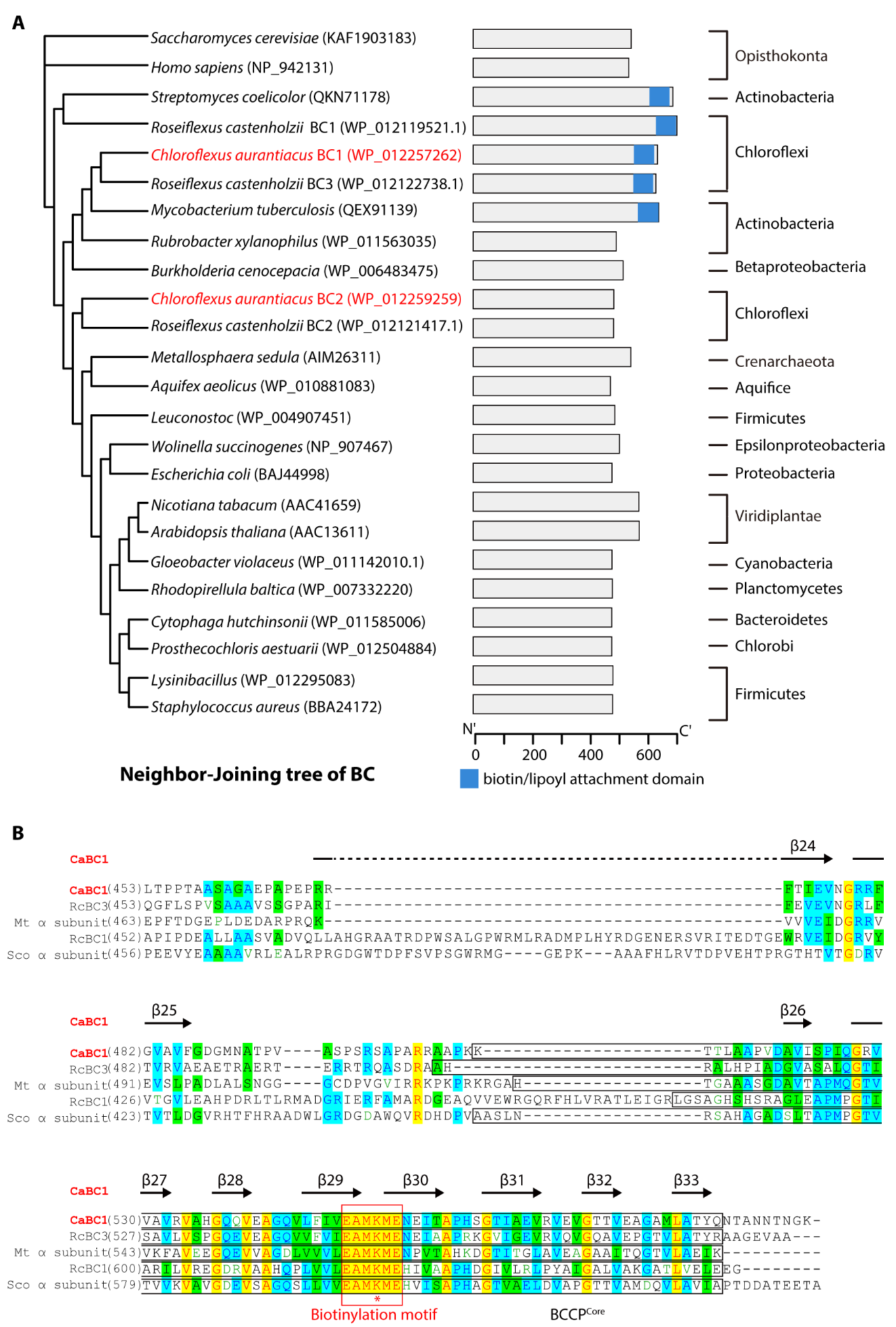
**

**FIG S1.** Phylogenetic analyses of biotin carboxylases (BCs) and multiple sequence alignment of the BC biotin/lipoyl attachment domains. (A) Phylogenetic tree of BCs. The neighbor-joining tree of BCs is reconstructed using neighbor-joining method, based on amino acid sequences of 24 BCs ranged from 15 kingdoms, and it is arbitrarily rooted on the ACC-related sequences. The biotin/lipoyl attachment domain is represented in dark blue colors. The ruler size represents the number of amino acids in the proteins. (B) Multiple sequence alignment of the BC biotin/lipoyl attachment domains from *Streptomyces coelicolor* (Sco), *Roseiflexus castenholzii* (Rc), *Chloroflexus aurantiacus* (Ca) and *Mycobacterium tuberculosis* (Mt). The amino acid residues possess identical and consensus sequences are highlighted in yellow, blue and green, respectively. The amino acid residues constitute the biotinylation motif and BCCP^Core^ are indicated with red and black boxes respectively. The essential lysine residue (K553) for biotinylation is marked with a red star. The secondary structures of the β hairpin and the C-terminal BCCP^Core^ predicted by Alphafold are labeled on the top of the sequence.


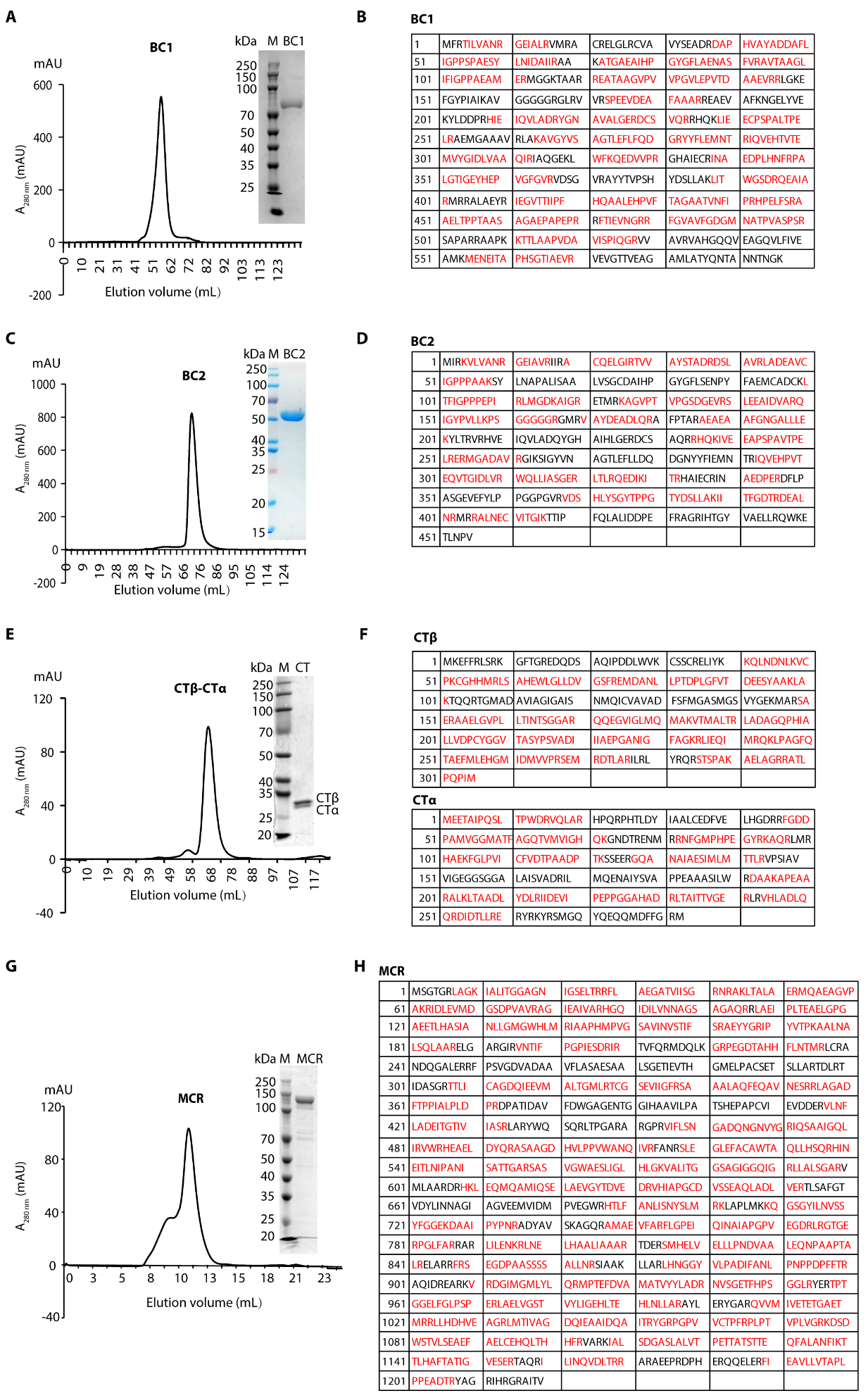


**FIG S2.** Gel filtration, SDS-PAGE and HPLC-ESI-MS/MS analysis of *Chloroflexus aurantiacus* BC1, BC2, carboxyltransferase (CT, CTβ-CTα) subcomplex and malonyl-reductase (MCR). (A, C, E, G) Gel filtration and SDS-PAGE of the recombinant BC1 (A), BC2 (C), CTβ-CTα (E) and MCR (G). The gel filtration profile depicts absorption at 280 nm (mAU) against elution volume (mL) of the proteins, which are eluted on a HiLoad 16/60 Superdex 200 pg column. SDS-PAGE indicates the purified BC1 (64 kDa), BC2 (50 kDa), CTβ (35 kDa), CTα (33 kDa) and MCR (132 kDa) eluted from the gel filtration column, respectively. (B, D, F, H) HPLC-ESI-MS/MS analysis of the recombinant BC1 (B), BC2 (D), CTβ and CTα (E), MCR (H). The amino acid residues detected by HPLC-ESI-MS/MS are colored in red.


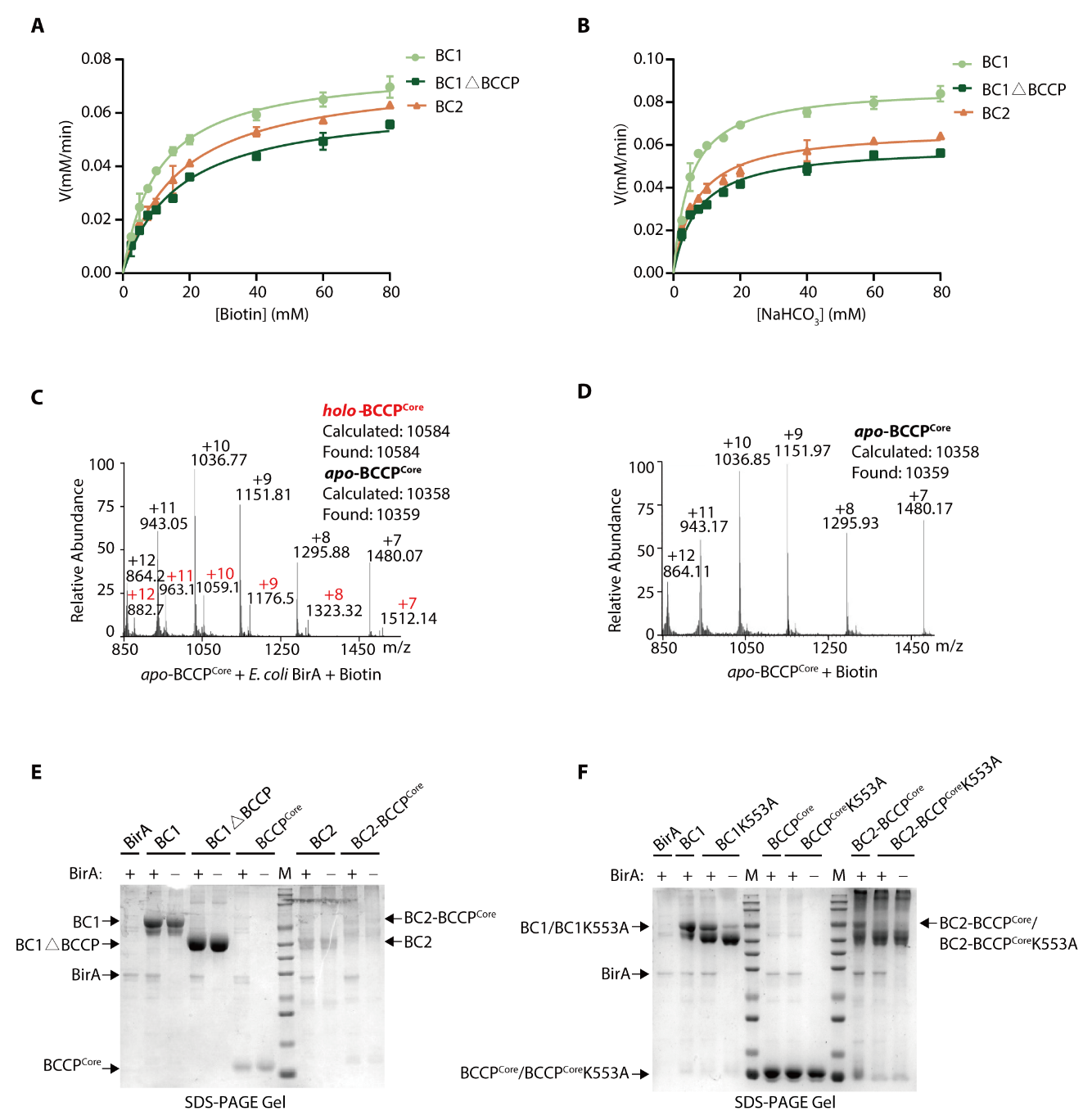


**FIG S3.** Biotin carboxylase and biotin carrier activity of *Chloroflexus aurantiacus* BC1, BC2 and the mutants. (A, B) Kinetic analyses of the biotin carboxylase activity of *C. aurantiacus* BC1, BC2 and BC1ΔBCCP when using biotin (A) and NaHCO_3_ (B) as the substrates. (C, D) HPLC-MS analyses to assess the biotinylation of the recombinant BCCP domain. (C) Biotinylated *holo*-BCCP was produced when *apo*-BCCP was incubated with biotin and *E. coli* BirA. (D) When BirA was absent in the reaction, no biotinylated *holo*-BCCP was detected. The calculated and found molecular weights of *holo*-BCCP were both 10584 Da. The calculated and found molecular weights of *apo*-BCCP were 10358 and 10359 Da, respectively. (E) Original SDS-PAGE of Figure 1D. (F) Original SDS-PAGE of Figure 1E. The protein bands corresponding to the BC1, BC2 and their mutants, and BirA were indicated with arrows. The presence and absence of BirA in the reactions was indicated with + and –, respectively.


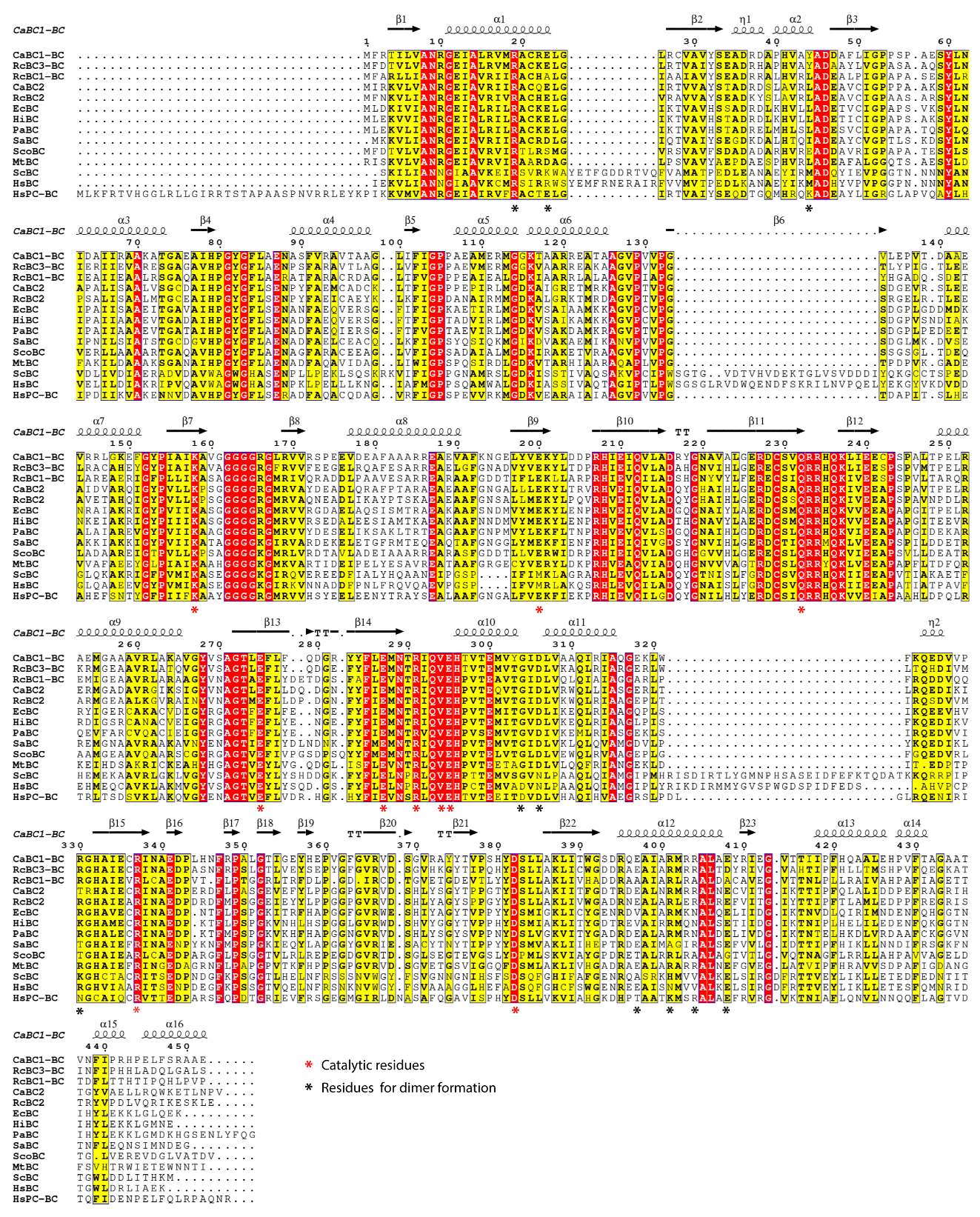


**FIG S4.** Structure-based sequence alignment of biotin carboxylases (BCs). Amino acid sequences of the BC subunits of heteromeric ACCs from *Escherichia coli* (Ec), *Haemophilus influenza* (Hi), *Pseudomonas aeruginosa* (Pa), *Chloroflexus aurantiacus* (Ca), *Roseiflexus castenholzii* (Rc), *Staphylococcus aureus* (Sa), *Streptomyces coelicolor* (Sco), *Mycobacterium tuberculosis* (Mt), and the BC subunits from *Saccharomyces cerevisiae* (Sc) homomeric ACC, *Homo sapiens* (Ho) homomeric ACC and pyruvate carboxylase (PC) are aligned. The secondary structures of *C. aurantiacus* BC1-BC are indicated on top of the sequence. The conserved catalysis residues are indicated with red stars, the conservative residues essential for dimer formation are indicated with black stars.

_
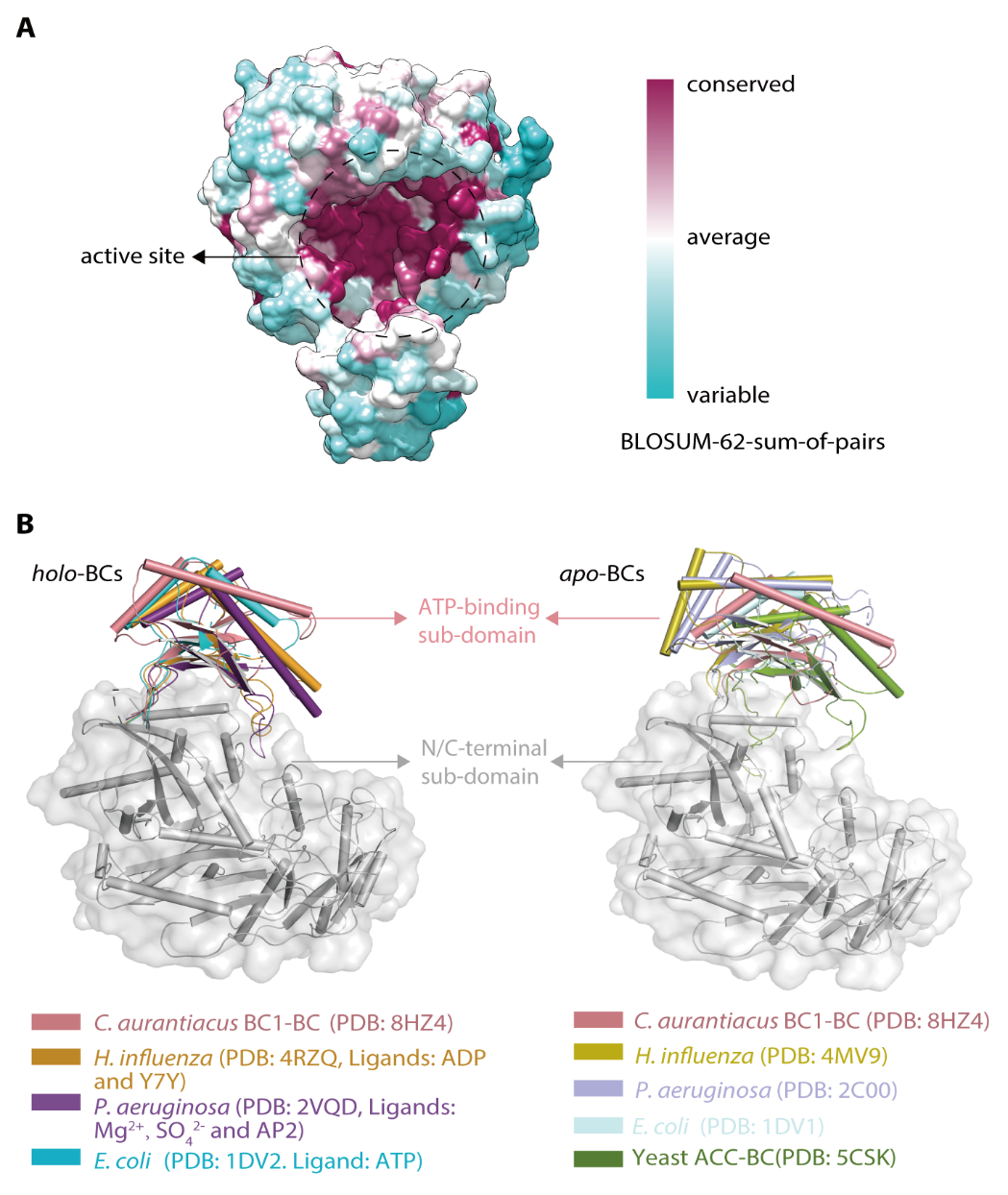
_

**FIG S5.** Structural conservations of the biotin carboxylases (BCs). (A) The sequence conservation of BCs is mapped onto the surface structure of BC1-BC monomer. Multiple sequence alignment of BCs from *E. coli*, *H. influenza*, *P. aeruginosa*, *C. aurantiacus*, *R. castenholzii*, *S. aureus*, *S. coelicolor*, *M. tuberculosis*, *S. cerevisiae* and *Homo sapiens* are performed using Clustal Omega. Then the monomer structure of BC1-BC is rendered by the sequence conservation using Chimera. The largely and completely conserved amino acid residues are shown in maroon, while the average conserved and variable residue positions are depicted in white and cyan, respectively. (B) Superposition of the *holo*-BC (left) and *apo*-BC (right) domains. The *apo*-BC subunits are from *C. aurantiacus* (salmon), *E. coli* (light blue), *H. influenza* (khaki), *P. aeruginosa* (light purple) and yeast (dark green), in which the N- and C-terminal subdomains are shown in surface and the ATP-binding sub-domains are shown in ribbon forms. Upon ligands binding, the ATP-binding sub-domains in *holo-*BCs from *E. coli* (cyan), *H. influenza* (dark yellow) and *P. aeruginosa* (purple) underwent dramatic conformational changes.


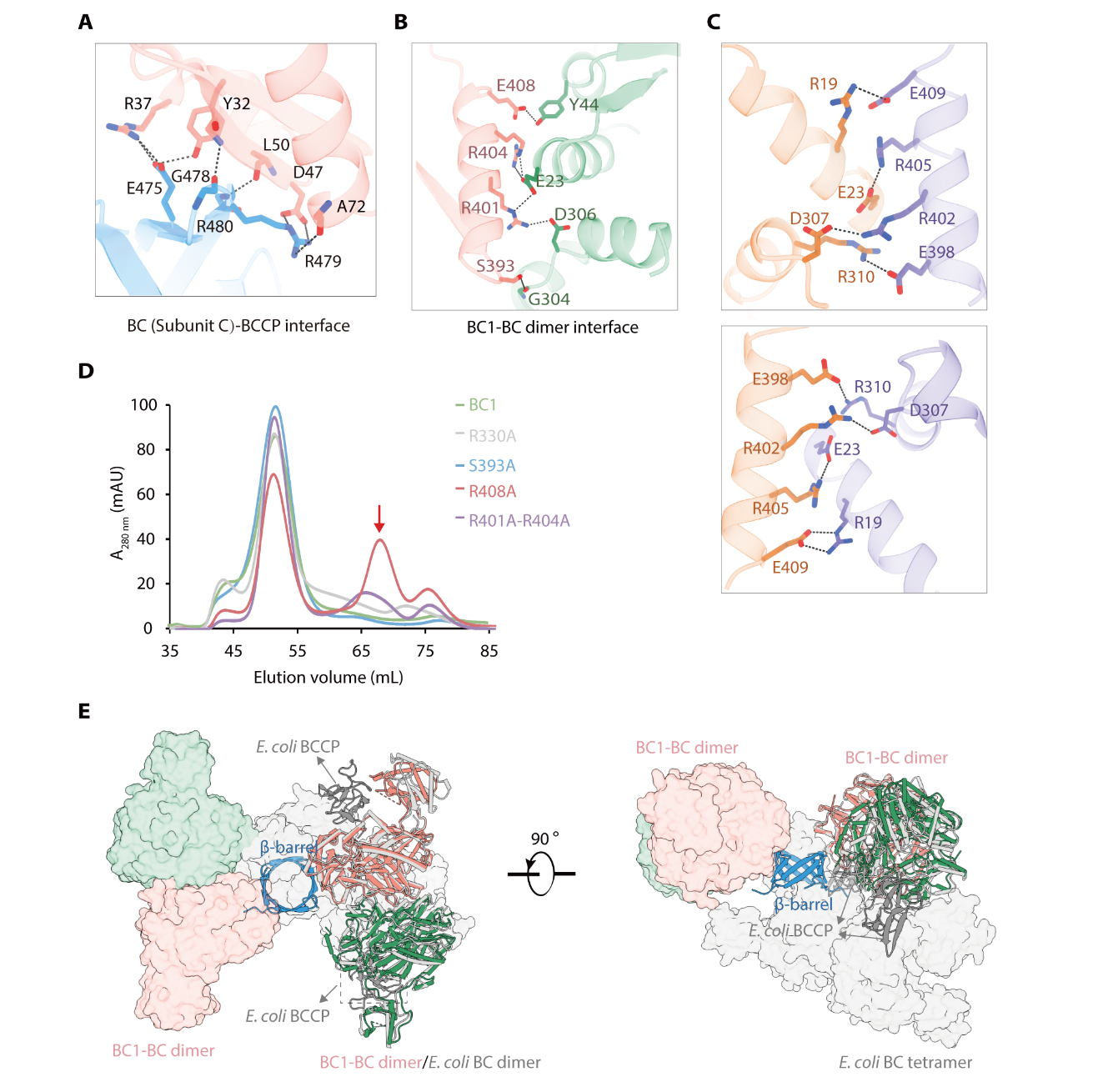


**FIG S6.** Crystal structures of *Chloroflexus aurantiacus* BC1, BC2 and gel filtration analyses of BC1 dimer interface mutants, and structural alignment. (A) Zoom in view of the BC (Subunit C)-BCCP dimer interface. The amino acid residues essential for mediating BC1-BC dimer interactions are shown in stick forms, and the hydrogen bonding interactions are indicated with dashed lines. (B) Zoom in view of the BC1-BC dimer interface. The amino acid residues essential for mediating BC1-BC dimer interactions are shown in stick forms, and the hydrogen bonding interactions are indicated with dashed lines. (C) Zoom in view of the BC2 dimer interface. The amino acid residues essential for mediating the dimer interactions are shown in stick forms, and the hydrogen bonding interactions are indicated with dashed lines. (D) Gel filtration analyses of BC1 dimer interface mutants. The gel filtration profile depicts the absorbance at 280 nm against elution volume (mL) from a HiLoad 10/300 Superdex 200 pg column. (E) Superposition of the crystal structure of BC1 tetramer onto the *E. coli* BCCP-BC octamer (PDB 4HR7). The colors of BC1-BC, BCCP, *E.coli* BC and *E. coli* BCCP were pink, marine, gray and yellow, respectively.


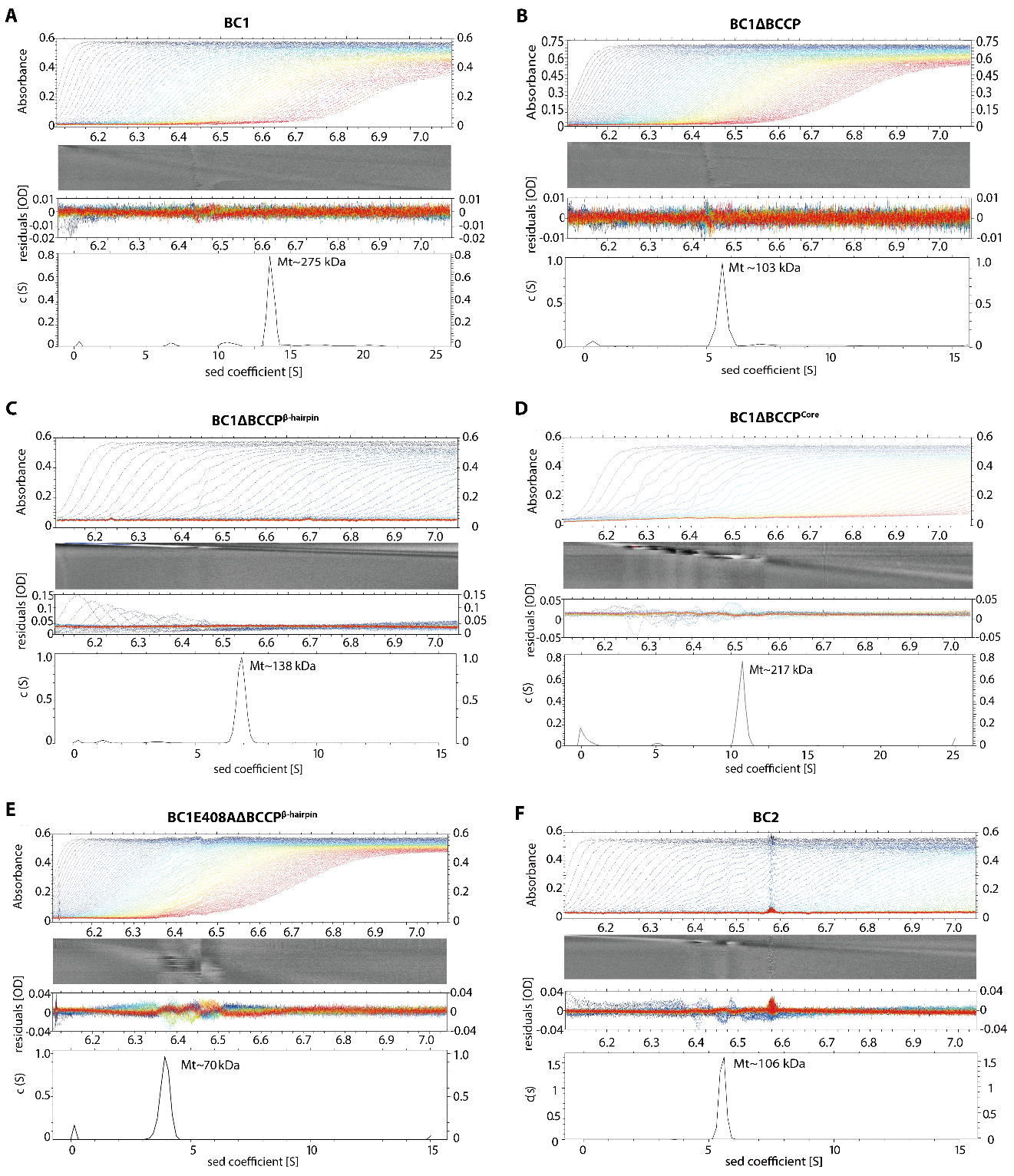


**FIG S7.** Sedimentation velocity analytical ultracentrifugation (AUC) analysis of *Chloroflexus aurantiacus* BC1 and mutants. (A-F) AUC analysis of BC1 (A), BC1ΔBCCP (B), BC1ΔBCCP^β-hairpin^ (C), BC1ΔBCCP^Core^ (D), BC1E408AΔBCCP^β-hairpin^ (E), and BC2 (F). The upper panel shows the raw data from the time-course measurement of absorbance at 280 nm along the sample cell length. The middle panel shows residuals after fitting the data to the continuous size-distribution model. The lower panel shows the continuous mass distribution for the calculated solution. The curve indicates that the BC1 (A) and BC1ΔBCCP^Core^ (D) exist as tetramers with ~275 and ~217 kDa molecular weight in solution respectively (the calculated MW of the monomer is 70.3 kDa). The BC1ΔBCCP (B), BC1ΔBCCP^β-hairpin^ (C), and BC2 (F) exist as homodimers of ~138, ~103 and ~106 kD in solution, and the BC1E408AΔBCCP^β-hairpin^ (E) exists as monomer of ~70 kD in solution. While the calculated MW of the monomer of BC1ΔBCCP, BC1ΔBCCP^β-hairpin^, BC1E408AΔBCCP^β-hairpin^ and BC2 is 54.4, 69.2, 69.2 and 54.9 kDa, respectively.


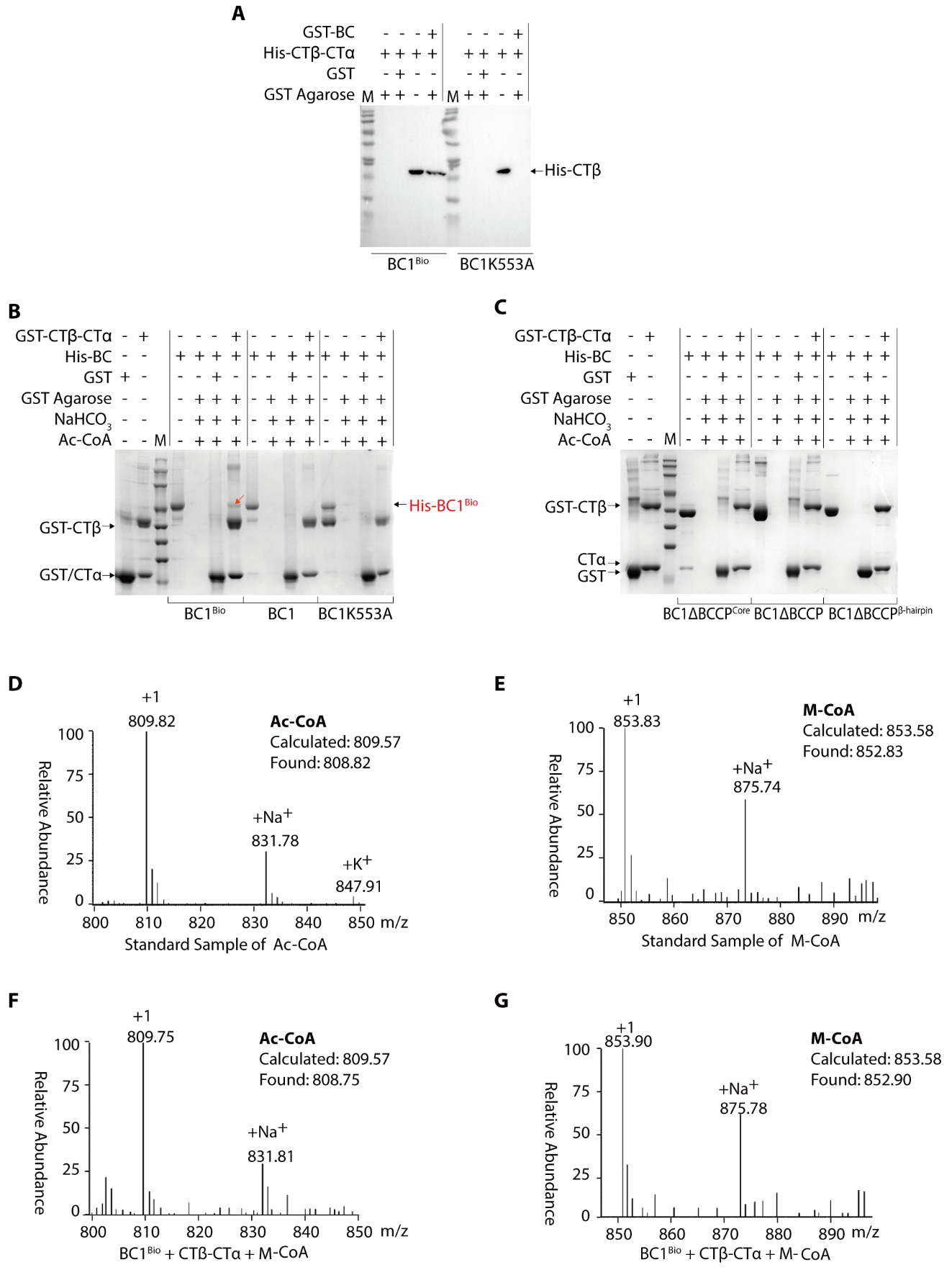


**FIG S8.** Biotinylated BCCP domain mediates direct interaction between BC1 and CTβ-CTα addition of substrates, and mass spectrometry analyses of the M-CoA and Ac-CoA produced by BC1 and CTβ-CTα mediated carboxylation reactions. (A) Western blot analysis of pull-down experiments between GST-CTβ-CTα and His_6_-tagged biotinylated BC1 (or BC1K553A). The protein bands were detected using His_6_-tag antibody. (B, C) Pull-down assays between GST-CTβ-CTα and His_6_-tagged biotinylated BC1, unbiotinylated BC1, BC1K553A (A), BC1ΔBCCP^β-hairpin^, BC1ΔBCCP, BC2-BCCP^Core^ (B) in the presence of substrates NaHCO_3_ and Ac-CoA. The proteins were separated using 10% SDS-PAGE, and the protein marker ladders ranging from 250 to 10 kDa are shown. + indicates the addition of the corresponding proteins listed on the left. (D) Detection of the Ac-CoA peak in Figure 5A with a retention time at 22.5-23.5 min. (E) Detection of the M-CoA peak in Figure 5A with a retention time at 6-8 min. (F, G) Detection of M-CoA and Ac-CoA peaks of reactions containing BC1^Bio^ + CTβ-CTα + M-CoA in Figure 5A with retention time of 6-8 min (F) and 22.5-23.5 min (G). The calculated and found molecular weights of Ac-CoA and M-CoA are indicated.


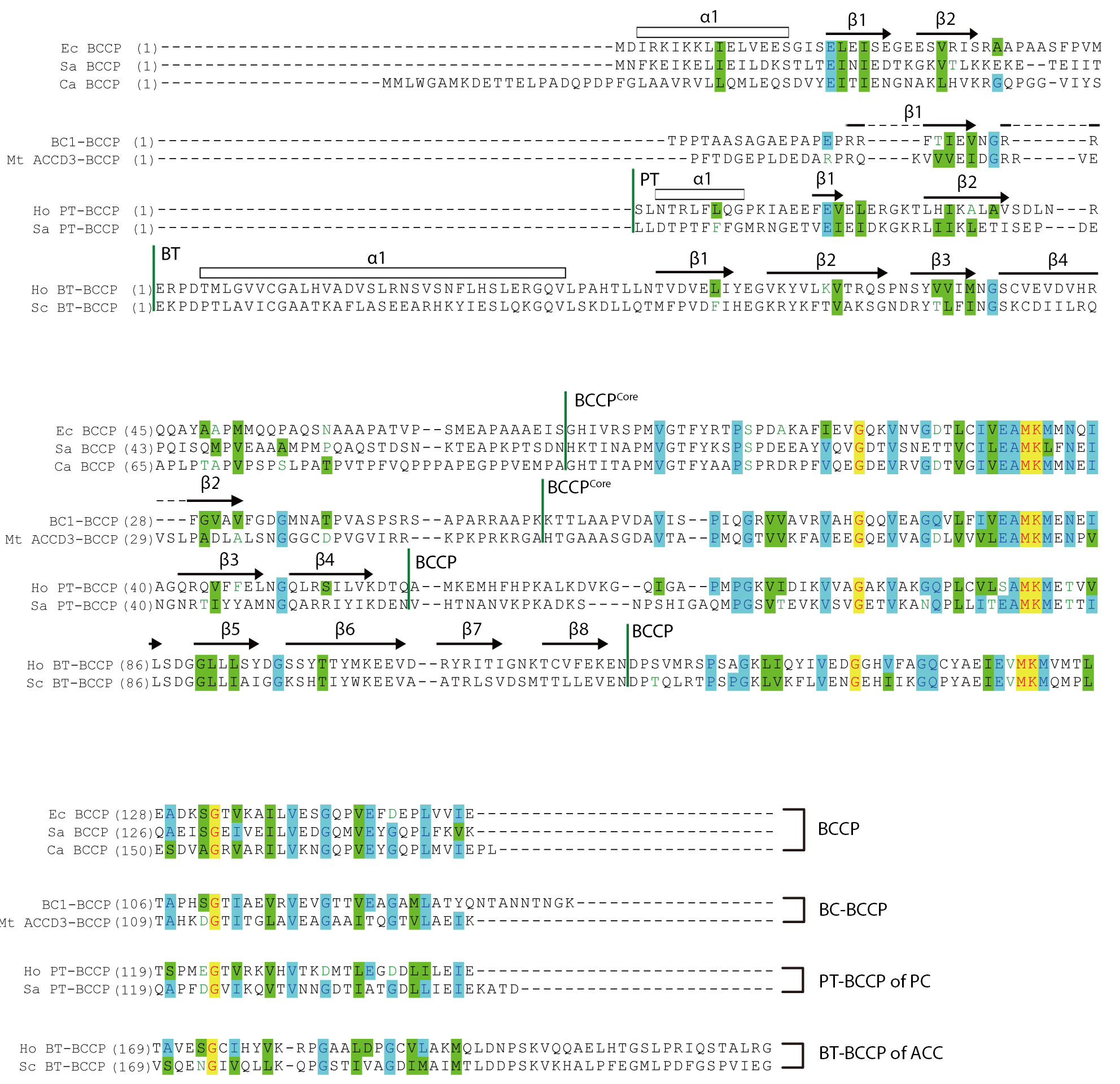


**FIG S9.** Multiple sequence alignment of the BCCP domains. The amino acid sequence of the BCCP domain from BC1 (BC1-BCCP) are aligned with the separate BCCP proteins from *C. aurantiacus* (Ca BCCP, Crystal structure), *Escherichia coli* (EC BCCP, Alphafold) and *Staphylococcus aureus* (Sa BCCP, Alphafold), and the fused BCCP domains from *Mycobacterium tuberculosis* AccD3 (Mt BCCP, Alphafold), *Staphylococcus aureus* (Sa) pyruvate carboxylase (PC) (PDB 3BG5), *Homo sapiens* PC (PDB 3BG3) and ACC and (PDB 6G2D), and *Saccharomyces cerevisiae* (Sc) ACC (PDB 5CSK). The amino acid residues possess identical and consensus sequences are highlighted in yellow, blue and green, respectively. The first amino acid residues constitute the single BCCP or BCCP^Core^, PT, BT are indicated with green line. The secondary structures are labeled on the top of the sequence.


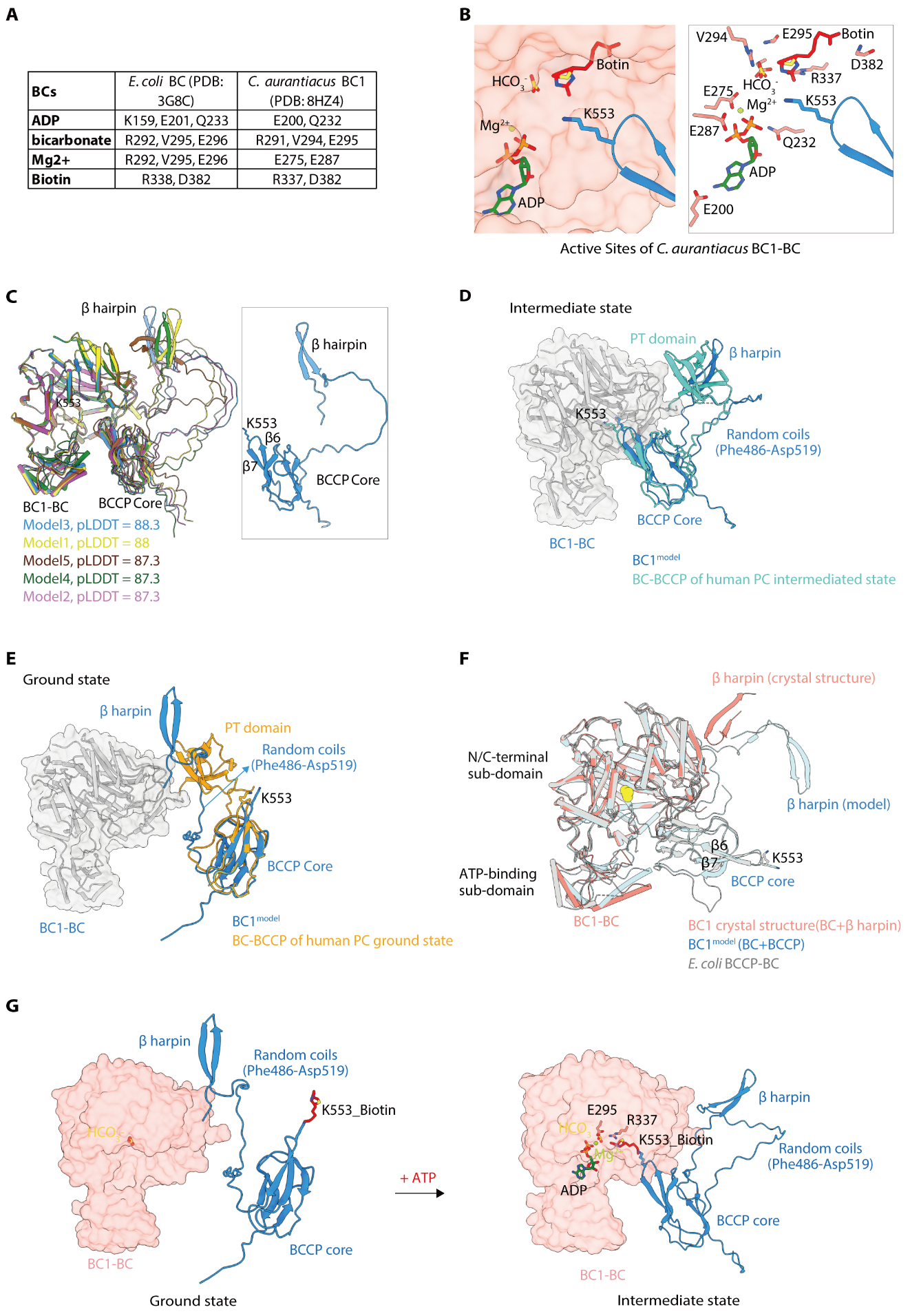


**FIG S10.** Structural basis of fused BCCP domain in mediating the biotin carboxylation activity of *Chloroflexus aurantiacus* BC1. (A) The amino acid residues involved in the binding of ADP, bicarbonate, Mg^2+^ and biotin from *E. coli* BC and *C. aurantiacus* BC1. (B) A magnified view of the substrate binding pocket of BC1-BC (pink) with modeled bicarbonate (yellow), Mg^2+^ (dark limon), ADP (green), and biotin (red). Key residues, including Lys553 and those amino acid residues essential for substrate binding, are shown as sticks. Hydrogen bonding interactions are illustrated with dashed lines. (C) Superposition of the simulated BC1 structural models. The BC1 structure containing complete BCCP domain was predicted by AlphaFold. Five models obtained with predicted local distance difference test (pLDDT) values above 87.3 are shown in cartoon forms. The Lys553 residue is shown in stick form. (D) The predicted BC1 model (blue) is superimposed onto the cryo-EM structure of the human PC intermediate state (PDB 7WTE, celadon). The biotinylated residue Lys553 is represented in stick form. (E) Superposition of the simulated BC1-BC and its BCCP domain (blue) onto the cryo-EM structure of human PC ground state (PDB 7WTC, khaki). (F) Superposition of the modeled BC1-BC and its BCCP domain (light blue) with the crystal structure of *C. aurantiacus* BC1 (pink), and crystal structure of *E. coli* BCCP-BC complex (PDB 4HR7, gray). (G) Proposed conformational changes of the fused BCCP domain during the biotin carboxylation reaction. In the ground state, the biotinylated Lys553 of the BCCP domain is positioned away from the active site of BC1-BC. Upon ATP binding, the BCCP domain undergoes conformational changes that translocates the BCCP^Core^ towards BC1-BC active site, within which the biotinylated Lys553 is carboxylated. During the reaction, Glu295 could initiate the ATP hydrolysis and Arg337 stabilizes the biotin-CO_2_^-^ intermediate. The biotin, Mg-ATP, bicarbonate, and amino acid residues Arg337 and Glu295 are depicted as sticks and labeled.
